# Benz[*c*,*d*]indolium-containing Monomethine Cyanine Dyes: Synthesis and Photophysical Properties

**DOI:** 10.3390/molecules21010023

**Published:** 2015-12-24

**Authors:** Eduardo Soriano, Cory Holder, Andrew Levitz, Maged Henary

**Affiliations:** 1 Department of Chemistry, Georgia State University, 50 Decatur St., Atlanta, GA 30303, USA; esoriano1@gsu.edu (E.S.); cholder1@gsu.edu (C.H.); alevitz1@gsu.edu (A.L.); 2 Center for Diagnostics and Therapeutics, Georgia State University, Petit Science Center, 100 Piedmont Ave SE, Atlanta, GA 30303, USA

**Keywords:** cyanine dye, unsymmetrical, synthesis, optical properties, DFT calculations, DNA grooves

## Abstract

Asymmetric monomethine cyanines have been extensively used as probes for nucleic acids among other biological systems. Herein we report the synthesis of seven monomethine cyanine dyes that have been successfully prepared with various heterocyclic moieties such as quinoline, benzoxazole, benzothiazole, dimethyl indole, and benz[*e*]indole adjoining benz[*c*,*d*]indol-1-ium, which was found to directly influence their optical and energy profiles. In this study the optical properties *vs.* structural changes were investigated using nuclear magnetic resonance and computational approaches. The twisted conformation unique to monomethine cyanines was exploited in DNA binding studies where the newly designed sensor displayed an increase in fluorescence when bound in the DNA grooves compared to the unbound form.

## 1. Introduction

Polymethine dyes represent a class of organic molecules with absorption bands that cover a broad spectral range (430–1100 nm), larger than any other class of dye system [1]. Cyanine dyes consist of two terminal aza-heterocycles connected via an electron deficient polymethine bridge that allows for a push/pull system between the two heterocycles. The delocalization of electrons across this bridge causes them to exhibit long wavelength absorptions. In addition to the variable length of the conjugated system between the heterocycles, the heterocycles themselves can be altered which allows chemists to create dyes that possess ideal photophysical properties, such as high molar extinction coefficients (>10^5^ M^−1^·cm^−1^), tunable fluorescence intensities, and narrow absorption bands. Due to the diversity in function associated with this class of chromophore, an extensive number of cyanine dyes have been synthesized and developed for numerous applications in photographic processes and more recently as fluorescent probes for bio-molecular labeling and imaging [1,2,3,4,5,6,7,8,9].

**Figure 1:**
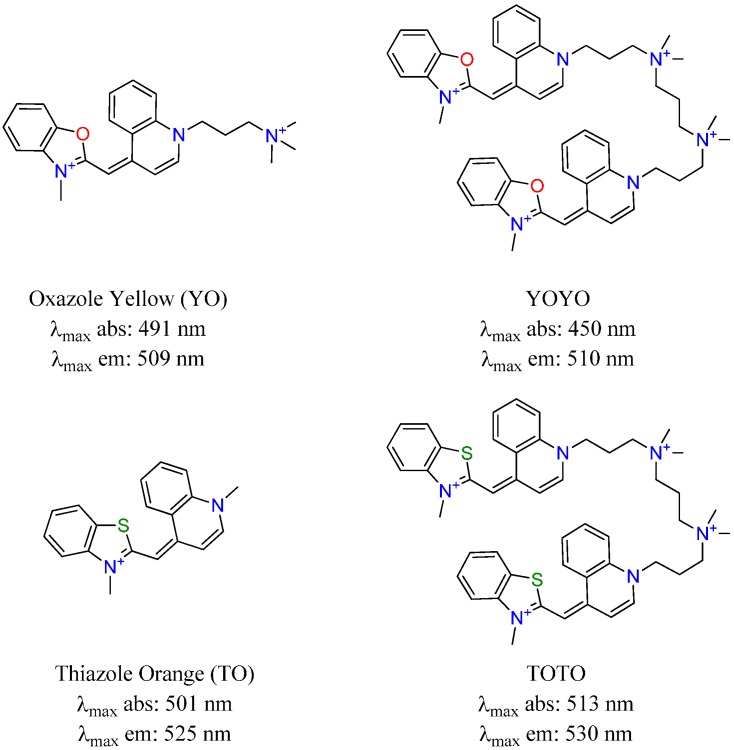
Commercially available asymmetric monomethine cyanine dyes.

As cyanine dyes have been shown to be highly modifiable for desirable properties such as solubility, permeability, and binding, these modifications can also cause changes in the dye’s photophysical properties. Recently, the interpretation of the fluorogenic behavior of the monomethine cyanine dyes from *in silico* studies has been successfully used to design new fluorescent molecular rotors as viscosity sensors [10]. Two asymmetric dyes shown in Figure 1, thiazole orange (TO) and oxazole yellow (YO), are well known imaging probes in the biological sciences due to their enhanced photophysical properties which have been attributed to restricted torsional motion of the dye in the excited state upon binding to target a macromolecule (*i.e.*, nucleic acid structure, protein) [11,12,13,14]. TO absorbs and fluoresces at 501 nm and 525 nm, respectively, while YO absorbs and fluoresces at 491 nm and 509 nm, respectively [15]. The dimers of these compounds are also known imaging probes and shown in Figure 1. YOYO absorbs and fluoresces at 450 nm [16] and 510 nm, respectively, while TOTO absorbs and fluoresces at 513 and 530 nm, respectively [17,18]. Nonetheless, there is a lack of understanding of how the structure interplays with the optical performance (*i.e.*, extinction coefficient and fluorescence)—especially for those monomethine cyanines with red-shifted wavelengths [9,19,20,21]. Thus, it is important to understand how varying substituents and heterocycles would affect the optical properties of each dye.

Imaging of macromolecules such as DNA by staining with fluorescent compounds is of great interest, therefore, expanding the options of available probes is vital to several areas of research spanning from medical diagnostics to genomics [22,23,24,25,26,27,28,29,30,31,32,33,34,35,36,37,38]. The synthesis of low cost, easy to manipulate systems for fast analysis is required [8]. Fluorescent detection has rapidly become one of the most widely used techniques due to its sensitivity and noninvasiveness [39]. Ethidium bromide has commonly been used for the detection of DNA, however it has mutagenic effects and poses other environmental concerns [40,41,42]. On the other hand, cyanine dyes are sensitive, safe and highly modifiable.

Recently, our group has synthesized a series of benz[*c*,*d*]indol-1-ium-containing monomethine cyanines with separate adjoining heterocyclic moieties which were found to directly influence the optical properties of the dye system [20]. In this report seven additional red-shifted monomethine cyanine dyes were synthesized and the structural influence on their fluorogenic properties was investigated by comparing the optical characteristics, examining the change in chemical shifts of methine proton and carbon NMR spectra, determining the energy profile through *in silico* approaches, as well as demonstrating that the dyes can be employed as DNA binding agents. The ability to use the theoretical calculations of optical properties for fluorophores, such as monomethine dyes could be useful for the development of the viscosity detection methods or bioimaging agents with desirable optical profiles.

## 2. Results and Discussion

### 2.1. Synthesis

Toward gaining better understanding of the relationship between various heterocyclic substitutions and changes in optical properties we began to rationally design and investigate the effect of altering the heterocyclic substitution on the photophysical characteristics of the dye systems. Two sets of monomethine cyanines were explored without altering the benz[*c*,*d*]indole heterocycle half of the dye. The first set possessing different heterocycles including 2-methylbenzothiazole, 2-methylbenzoxazole, 3,3-dimethylbenz[*e*]indole or 2-methylquinoline, respectively, and the second set containing the same 3,3-dimethylindole heterocycle, but with different substituents, one electron donating and one electron withdrawing, on the 5 position of the heterocyclic ring system.

**Scheme 1:**
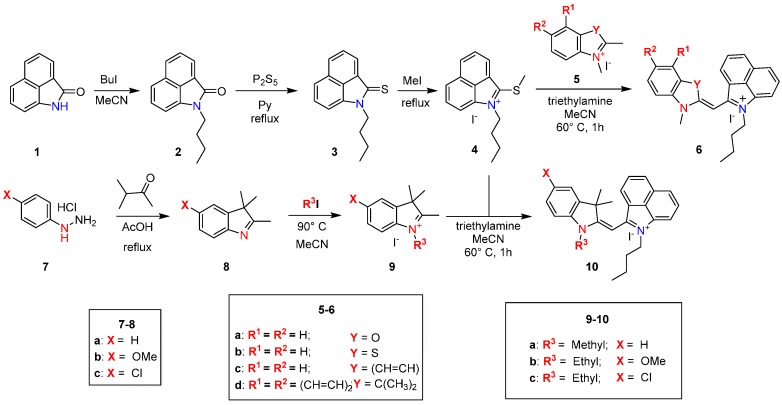
Synthesis of Monomethine Dyes.

The asymmetric red-shifted monomethine cyanine dyes were synthesized as shown in Scheme 1. The synthesis began with the alkylation of benz[*c*,*d*]indol-2(1*H*)-one (**1**) by reflux with iodobutane in acetonitrile. The alkylated amide **2** was then converted to the thioketone **3** under reflux with phosphorous pentasulfide in pyridine. The thioketone **3** was methylated to a thioether with iodomethane creating the key precursor, quaternary ammonium salt **4**, which was used as one heterocycle. The second heterocycle was synthesized beginning with a Fischer indole synthesis by refluxing 4-substituted phenylhydrazine hydrochlorides **7** and 3-methyl-2-butanone in glacial acetic acid. The synthesized heterocyclic derivatives **8**, 2-methylbenzothiazole, 2-methylbenzoxazole, 2,3,3-trimethylbenz[*e*]indole, and 2-methylquinoline were alkylated, respectively, with various alkyl halides in acetonitrile to form quaternary ammonium salts **5a**–**d** and **9a**–**c**, which acted as the second heterocycle for the final dyes. The two heterocycles were then connected by a condensation reaction in acetonitrile with a catalytic amount of triethylamine to afford final dyes **6a**–**d** and **10a**–**c**.

The reaction begins with the deprotonation of the methyl group at the 2 position of the heterocycle. This activated methylene group of the various heterocyclic salts **5a**–**d** and **9a**–**c** displaces the methyl sulfide moiety of **4** and results in the formation of the asymmetrical monomethine dyes **6a**–**d** and **10a**–**c**. After isolation, the dyes were characterized by HRMS, ^1^H- and ^13^C-NMR and their photophysical properties were investigated.

### 2.2. Optical Properties

**Figure 2:**
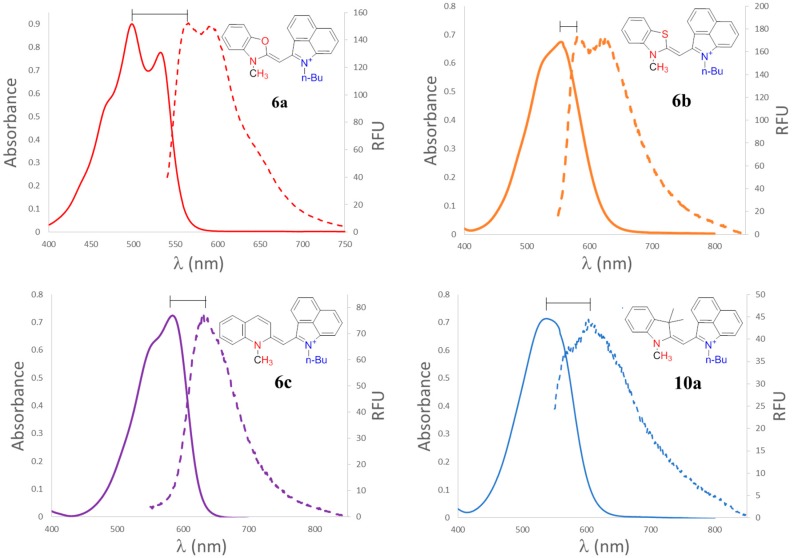
Absorbance (solid lines) and emission (dashed lines) in methanol/glycerol 9/1 spectra at 20 μM.

Optical properties are shown in Table 1. Absorption for each dye was recorded in methanol and 9/1 glycerol/methanol solution. Many monomethine cyanines display multiple bands which are attributed to different vibronic bands of the same electronic transition [16]. Because the compounds did not fluoresce in methanol due the ability to freely rotate around the methine bridge in free flowing solvent, emission was recorded in a more viscous solvent, 9/1 glycerol/methanol solution. Representative UV-Vis spectra are shown in Figure 2. A symmetrical monomethine dye containing two benzothiazole heterocycles has a λ_max_ of 430 nm in ethanol [43]. It has been shown by Brooker *et al.*, that if the nitrogen containing heterocycles are not identical, or if the relative stabilities of the two resonance forms are different, the absorption would not be at the midpoint [44]. The substitution of one of these heterocycles with benz[*c*,*d*]indole shifts the λ_max_ over 100 nm to 555 nm as seen in **6b**. This was accounted for by the further conjugated electron deficient system in the benz[*c*,*d*]indole heterocycle [1,20,45,46]. The conjugated system has more electronegativity due to the oxygen atom in **6a** causing a blue shift of the λ_max_ to 498 nm [11,47]. While the compounds containing 3,3-dimethylindole have similar absorption maxima to the benzothiazole compounds, the addition of an extra benzene ring as seen in **6d** red shifts the λ_max_ to 585 nm due to the increased conjugation through the heterocycle. All of the dyes displayed molar extinction coefficients in the range of 24,000–38,000 M^−1^·cm^−1^. The dye with a methoxy substituted indole heterocycle **10b** showed the lowest molar absorptivity at 24,800 M^−1^·cm^−1^ due to the electron donating nature of the methoxy group introducing electron density back into the system [47,48]. Aggregation was ruled out by measuring absorption of **6b** as a representative compound at various concentrations (5–25 μM) and the results were presented in the Supplementary Materials (Figure S2G). Solvatochromic studies were performed on dye **6b** in five different solvents (ethanol, dimethyl formamide, dichloromethane, acetonitrile, and aqueous tris buffer) (Figure S2H). Less than 5 nm change in λ_max_ was observed. Such a small shift suggests that the electronic distribution of the ground state dye is virtually unaffected by the solvent polarity [47].

**Table 1:** Spectral Characteristics of Dyes **6a**–**d** and **10ac**.

Dye	λ_abs_ (nm) ^a^	λ_abs_ (nm) ^b^	λ_emission_ (nm) ^b^	Stokes Shift (nm) ^b^	ε (M^−1^·cm^−1^) ^a^
**6a**	498	505	570	65	37600
**6b**	555	563	609	46	32300
**6c**	585	587	609	22	36500
**6d**	553	557	625	68	25300
**10a**	537	552	657	105	33300
**10b**	563	569	606	37	24800
**10c**	552	571	662	91	30100

^a^ methanol ^b^ methanol/glycerol 9/1 (*v*/*v*).

It has been reported that the fluorescence of these compounds cannot be observed in methanol alone because of a high nonradiative rate of return of the excited molecule as previously reported with many monomethine cyanines [20,49,50,51]. However, when a viscous solution is used, the free rotation around the methine bridge is restricted and a fluorescence signal is observed as shown in Figure 2. Methanol (10%) was used in order to solubilize the compounds in the highly viscous glycerol. Fluorescence maxima ranged from 570 nm to 662 nm, almost reaching the near-infrared region. The benzoxazole containing dye **6a** had the highest fluorescence intensity followed by benzothiazole containing dye **6b**. The quinoline containing dye **6c** had the least fluorescence intensity due to alternative relaxation pathways [52]. The largest Stokes shift, greater than 100 nm, was observed for the dye with an indole based heterocycle, **10a**. Since the emission intensity was so low the Stokes shift reported could be slightly skewed due to low signal to noise. However, this finding is in agreement with red-shifted compounds previously synthesized by our group [20]. Large Stokes shifts are ideal for imaging applications as the excitation light is farther from the fluorescence signal of the compound [39,53].

### 2.3. Computational Evaluations

**Figure 3:**
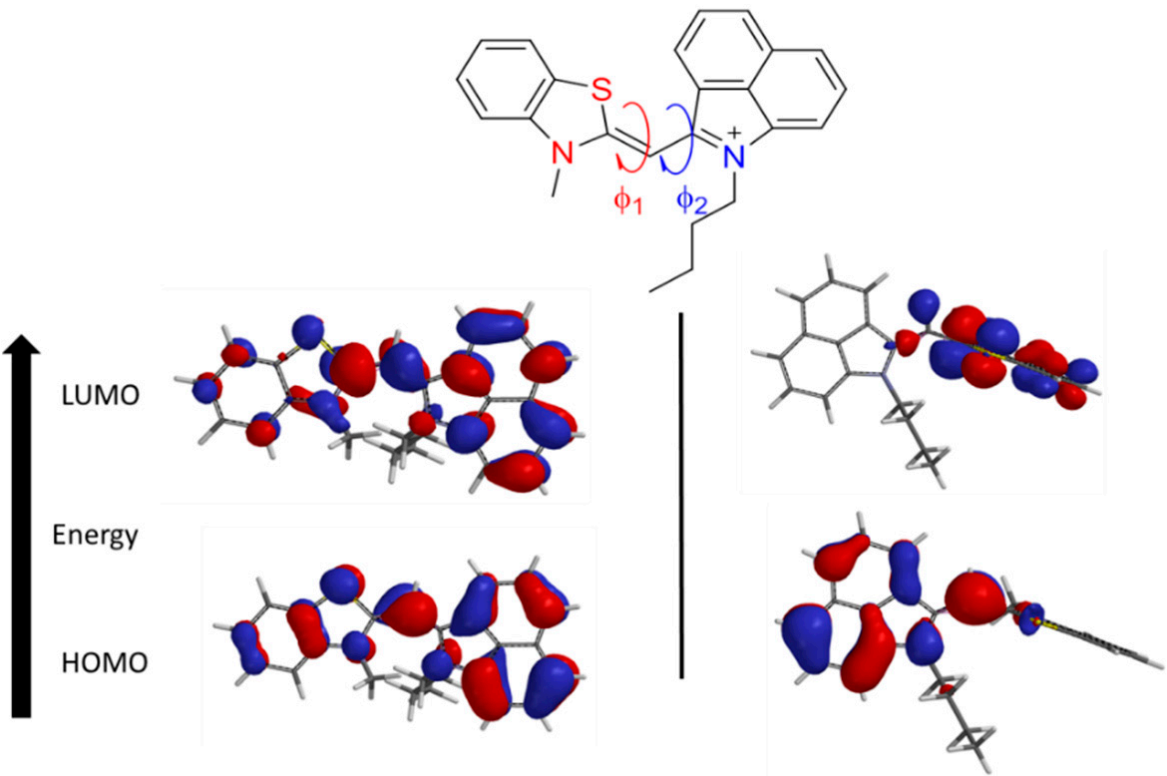
Frontier molecular orbitals of **6a** constrained in planar (**left**) and twisted (**right**) configurations.

The electronic spectra of the monomethine dyes were investigated to help elucidate the trends described above in the optical properties. As shown by the calculations in Figure 3, over the series of dyes when the geometry is planar both the HOMO and LUMO orbitals are spread evenly throughout the dye. When the dyes are twisted out of plane the HOMO orbitals are localized around the more conjugated system benz[*c*,*d*]indole heterocycle. The energy transitions in cyanine dyes have been shown to be a dominant π–π* transition [11,21], but if the dye assumes a twisted geometry the orbitals are not delocalized throughout the dye, as shown in Figure 3, and the system is not conjugated or planar [54].

**Figure 4:**
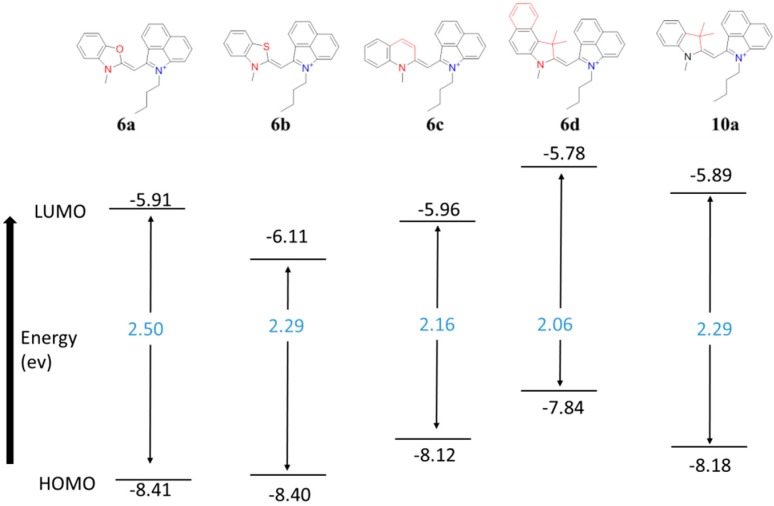
HOMO and LUMO orbital analysis of differing heterocycles in the monomethine system; energies (black), HOMO-LUMO gaps (blue).

The geometry was constrained to keep the molecule planar to observe trends in the HOMO–LUMO gaps for comparing with excitation energies. As shown in Figure 4, the energy gap between HOMO and LUMO of compound **6d** containing a benz[*e*]indole heterocycle is the lowest among the series of dyes at 2.06 eV. This finding is corroborated by the bathochromic absorbance maximum of the benz[*e*]indole compared to the Fischer indole, benzothiazole, and benzoxazole heterocycles which led to further delocalizing of the electrons and therefore stabilizing the orbitals.

The benzoxazole heterocycle in dye **6a** influenced the conjugated system shown by shifting the absorbance maximum to the blue. This dye **6a** shows the highest energy gap likely due to both the lone pair of electrons and electronegativity of the oxygen atom similar to dye **6b** with a sulfur containing benzothiazole heterocycle that had the second highest energy gap. Dye **10a** containing a 3,3-dimethylindole heterocycle had the same energy gap as **6b** with the benzothiazole heterocycle, but had higher energy.

**Figure 5:**
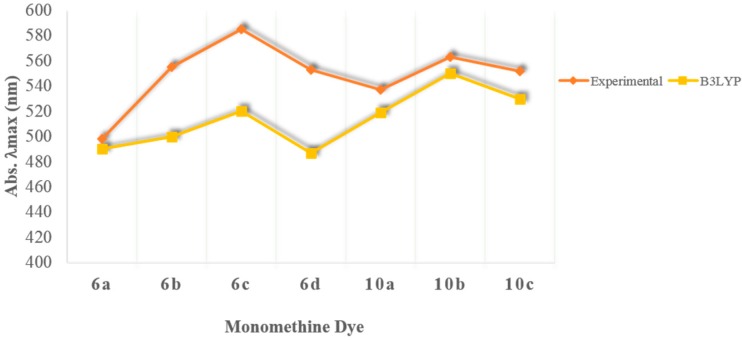
Experimental and Calculated λ_max_ values.

The theoretical absorption λ_max_ values are plotted along with the experimental data as shown in Figure 5. Time-dependent density functional theory (TD-DFT) has been shown to work well for large conjugated molecules because the orbitals are obtained by solving the Kohn-Sham equation involving exchange and correlation (XC) terms [55]. Although a discrepancy gap is observed between the theoretical and experimental results, the observed trends in absorbance wavelength are almost the same with the calculated absorbance wavelength giving slightly blue-shifted values [47].

**Figure 6:**
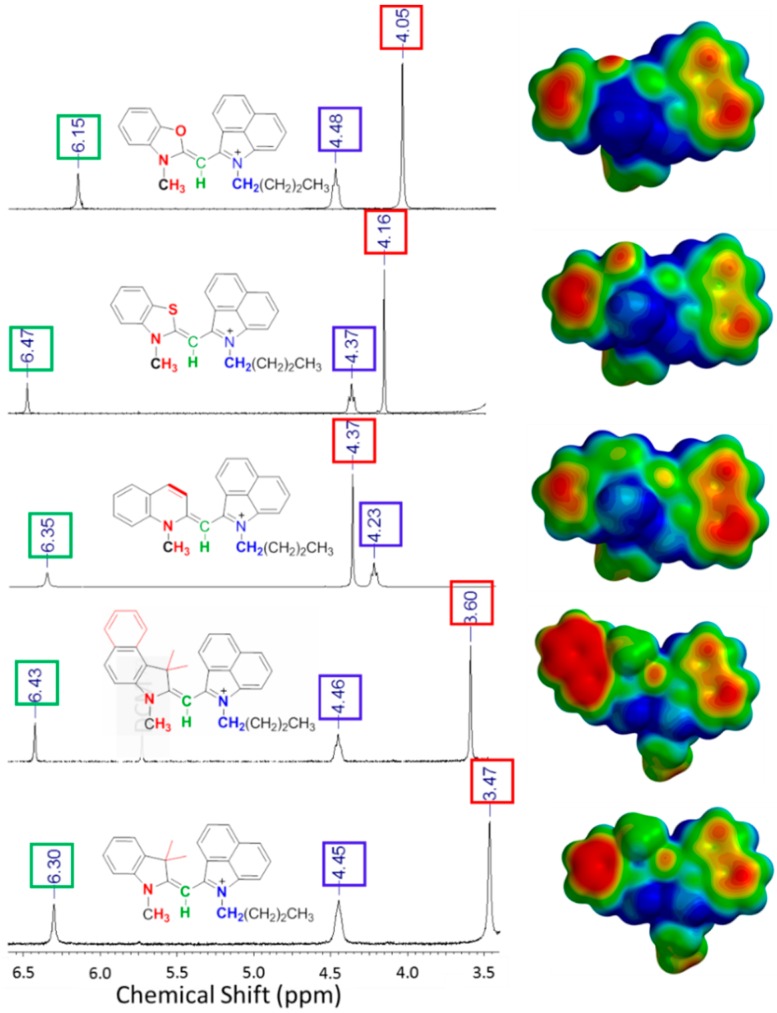
^1^H-NMR shift of *meso*-proton in DMSO-*d*_6_ at 25 °C, Calculated EMP on the right.

As shown in Figure 6 and Table 2, the observed change of the chemical shift of the methine-proton is most likely due to altering the electron density from the surrounding atoms.

**Table 2:** λ_max_, NMR shifts, and computational charges of monomethine cyanine dyes.

	Heterocycle Included in Monomethine Dye	λ_abs_ (nm) exp.	λ_abs_ (nm) calc.	Charge of Methine Carbon	Methine Carbon Shift (ppm)	Methine Proton Shift (ppm)	N-CH_3_^1^H Shift (ppm)
**6a**	benzoxazole	498	490	−0.535	75.51	6.15	4.05
**6b**	benzothiazole	555	500	−0.421	87.40	6.47	4.16
**6c**	quinoline	585	520	−0.526	93.65	6.35	4.37
**6d**	benz(e)indole	553	487	−0.284	94.10	6.43	3.60
**10a**	3,3-dimethylindole	542	519	−0.328	82.78	6.30	3.47
Substitution at the 5-position of heterocycle **10a**							
**10a**	H	542	519	−0.328	82.78	6.31	-
**10b**	OMe	563	550	−0.316	83.44	6.23	-
**10c**	Cl	552	530	−0.344	83.81	6.29	-

Calculated values obtained via TD-DFT in vacuum, NMR run in DMSO-*d_6_* at 25 °C.

### 2.4. DNA Binding

**Figure 7:**
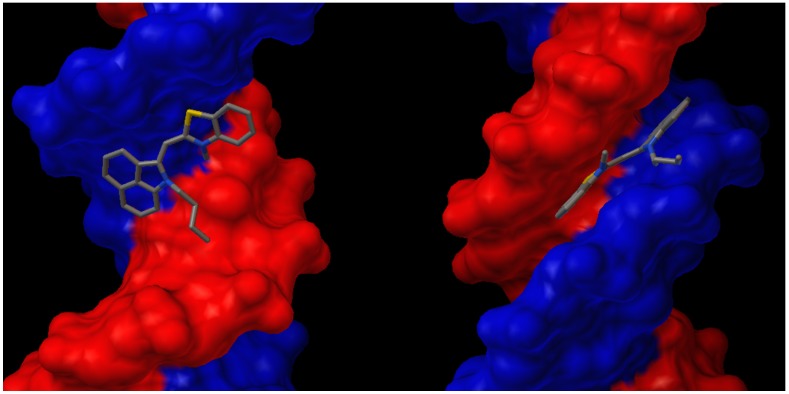
Dye **6b** with fixed torsion angles and planar geometry suggested to bind to the major (**left**) and minor (**right**) grooves of dsDNA by computational studies.

It has been reported that a combination of a crescent shape complements the helical DNA minor groove, hydrogen bond donors and acceptors on the side of the molecule facing the DNA, a cationic center to enhance electrostatic interactions with negatively charged phosphate groups, and hydrophobic character from an extended fused heterocyclic structure allows for optimization of the compound for DNA minor groove interactions [56,57,58,59]. Dye **6b**, which is crescent shaped and has an overall hydrophobic structure, includes a sulfur on the side suggested by computational data to be facing the DNA (Figure 7) and contains delocalized positive charge throughout the polymethine chain; therefore, it was selected for DNA binding as a representative example of the series.

**Figure 8:**
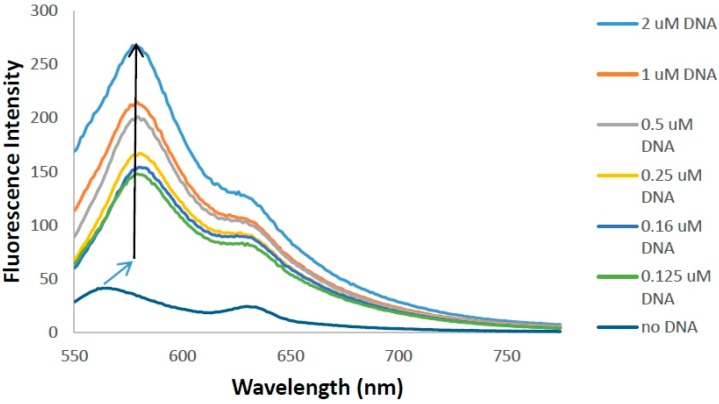
Emission spectra of dye **6b** (10 μM) in Tris-HCl buffer with and without ct-DNA (excitation wavelength 520 nm).

As presented in Figure 8, the fluorescence spectrum of **6b** in Tris-HCl buffer exhibits a particularly weak fluorescence spectrum with 2 local maxima at 565 nm and 630 nm. The 565 nm band is red shifted to 582 when ct-DNA is added and an increase in fluorescence is observed. Similar to the previously described enhancement in glycerol, a viscous solvent, this enhancement is also attributable to the fact that on excitation the inability to freely rotate around the methine bond due to binding does not allow for nonradiative deactivation of the ground state causing the dye to fluoresce. Using a double reciprocal plot, the binding constant, *K*_b_, of **6b** was determined to be 1.0 × 10^4^ M^−1^ which is on par with similar monomethine cyanine dyes [8].

Although dye **6b** is structurally similar to TO (Figure 1), a known intercalating agent, it is intriguing to investigate interactions at the molecular level. Therefore, computational studies were conducted to get better insight on the mode of binding for these red shifted monomethines. The 264D (a dodecamer d(CGCAAATTTGCG)_2_) was chosen from the Protein Data Bank as a representative model for dsDNA binding. Molecular docking was then performed on **6b** using Autodock (Figure 7). As it turns out, docking was achieved in both the minor and major grooves. Our computational data indicates higher propensity to bind in the minor groove based on relative scoring. Surprisingly, **6b** did not display intercalation based on these computational studies. This could be due to the bulkiness of the benz[*c*,*d*]indole heterocycle. Further studies such as electrophoresis unwinding assays or crystallography can be conducted in the future to more accurately define the binding modes of these compounds.

## 3. Experimental

### 3.1. General Information

All chemicals and solvents were of American Chemical Society grade or HPLC purity and were used as received. HPLC grade methanol and glycerol were purchased from Sigma-Aldrich (St. Louis, MO, USA). All other chemicals were purchased from Fisher Scientific (Pittsburgh, PA, USA) or Acros Organics (Pittsburgh, PA, USA). The reactions were followed using silica gel 60 F_254_ thin layer chromatography plates (Merck EMD Millipore, Darmstadt, Germany). The ^1^H-NMR and ^13^C-NMR spectra were obtained using high quality Kontes NMR tubes (Kimble Chase, Vineland, NJ, USA) rated to 500 MHz and were recorded on an Avance spectrometer (Bruker, Billerica, MA; 400 MHz for ^1^H and 100 MHz for ^13^C) in DMSO-*d*_6_ or CD_3_Cl-*d*_3_. High-resolution accurate mass spectra (HRMS) were obtained at the Georgia State University Mass Spectrometry Facility using a Q-TOF micro (ESI-Q-TOF) mass spectrometer (Waters, Milford, MA, USA). HPLC data was obtained using a Waters 2487 dual detector wavelength absorption detector with wavelengths set at 260 and 600 nm. The column used in LC was a Waters Delta-Pak 5 μM 100 Å 3.9 × 150 mm reversed phase C18 column, with a flow rate of 1mL/min employing a 5%–100% acetonitrile/water/0.1% formic acid gradient. All compounds tested were >95% pure. Infrared spectra (FT-IR) were obtained using a Spectrum 100 spectrometer (PerkinElmer, Duluth, GA, USA) (see Supplementary Materials). UV-Vis/NIR absorption spectra were recorded on a Cary 50 spectrophotometer (Varian, Palo Alto, CA, USA) interfaced with Cary WinUV Scan Application v3.00 using VWR disposable polystyrene cuvettes with a 1 cm pathlength. Laser Induced Fluorescence (LIF) emission spectra were acquired using Shimadzu RF-5301 Spectroflurophotometer (Shimadzu Corporation Analytical Instruments Division, Duisburg, Germany) interfaced to a PC with RF-5301PC software using Sigma-Aldrich disposable polystyrene fluorimeter cuvettes with a 1 cm pathlength. All spectral measurements were recorded at room temperature. The data analysis and calculations were carried out using Microsoft Excel (Microsoft Corporation, Redmond, WA, USA).

### 3.2. Synthesis

#### 3.2.1. General Synthetic Procedure for the Indolium Salts **4** and **9a**–**c**

Thioether **4** was previously synthesized by our group and others [20,60]. The substituted indoles **8** were synthesized as previously reported by our group and others [20,61]. Each individual compound **8** was dissolved in acetonitrile and alkyl halide was added. The reaction mixture was then refluxed for 12 h. Thin layer chromatography (TLC) was used to monitor the reaction progress using a mixture of 4:1 dichloromethane-hexanes. Upon cooling to room temperature, the quaternary ammonium salts **9a**–**c** were precipitated in diethyl ether and collected by vacuum filtration [36,62].

#### 3.2.2. General Synthesis of the Monomethine Dyes

Thioether **4** and each quaternary ammonium salt **5a**–**d** and **9a**–**c**, respectively, were dissolved in acetonitrile and a catalytic amount of triethlyamine was added to the solution. The reaction mixture was refluxed at 60 °C for 1 h and monitored by UV-Vis*.* Upon cooling to room temperature, the corresponding dyes **6a**–**d** and **10a**–**c** were precipitated by adding diethyl ether. The solid was collected by vacuum filtration and triethylammonium salts were removed by washing with deionized water. The final dyes were purified via precipitation from methanol with diethyl ether.

*1-Butyl-2-[(3-methyl-1*,*3-benzoxazol-2(3H)-ylidene)methyl]benzo[c*,*d]indolium iodide* (**6a**); Yield 0.43 g, 69%; mp > 260 °C; ^1^H-NMR (DMSO-*d*_6_): δ ppm 0.95 (t, *J* = 7.1 Hz, 3H), 1.44–1.49 (m, 2H), 1.82–1.85 (m, 2H), 4.04 (s, 3H), 4.48 (t, *J* = 7.3 Hz, 2H), 6.14 (s, 1H), 7.55–7.67 (m, 3H), 7.73 (t, *J* = 8.6 Hz, 1H), 7.82–7.89 (m, 2H), 8.04 (t, *J* = 7.3 Hz, 1H), 8.15 (d, *J* = 7.1 Hz, 1H), 8.39 (d, *J* = 7.6 Hz, 1H), 9.17 (d, *J* = 7.6 Hz, 1H); ^13^C-NMR (DMSO-*d*_6_): δ ppm 14.3, 20.1, 30.2, 32.0, 75.5, 110.1, 112.3, 112.6, 126.6, 127.2, 129.7, 129.7, 130.3, 130.4, 131.8, 132.9, 141.1, 146.8, 155.6, 162.0; HRMS (ESI): Calcd for C_24_H_23_N_2_O^+^ *m*/*z* 355.1805, obsd *m*/*z* 355.1791.

*1-Butyl-2-[(3-methyl-1*,*3-benzothiazol-2(3H)-ylidene)methyl]benzo[c*,*d]indolium iodide* (**6b**); Yield 0.37 g, 57%; mp 249–251 °C; ^1^H-NMR (DMSO-*d*_6_): δ ppm 0.96 (t, *J* = 7.3 Hz, 3H), 1.43–1.49 (m, 2H), 1.75–1.92 (m, 2H), 4.16 (s, 3H), 4.37 (t, *J* = 7.2 Hz, 2H), 6.47 (s, 1H), 7.55 (d, *J* = 7.3Hz, 1H), 7.59–7.72 (m, 2H), 7.74–7.81 (m, 2H), 7.89 (t, *J* = 7.8 Hz, 1H), 8.04 (d, *J* = 8.3 Hz, 1H), 8.20 (d, *J* = 7.8 Hz, 1H), 8.32 (d, *J* = 8.1 Hz, 1H), 9.25 (d, *J* = 7.6 Hz, 1H); ^13^C-NMR (DMSO-*d*_6_): δ ppm 13.8, 19.7, 29.7, 35.4, 43.4, 87.0, 109.0, 115.0, 122.0, 123.6, 124.7, 126.8, 128.8, 129.2, 129.6, 129.7, 132.3, 141.0, 141.2, 154.0, 165.9; HRMS (ESI): Calcd for C_24_H_23_N_2_S^+^ *m*/*z* 371.1576, obsd *m*/*z* 371.1566.

*1-Butyl-2-[(1-methylquinolin-2(1H)-ylidene)methyl]benzo[c*,*d]indolium iodide* (**6c**); Yield 0.44 g, 69%; mp 225–227 °C; ^1^H-NMR (DMSO-*d*_6_): δ ppm 0.95 (t, *J* = 7.2 Hz, 3H), 1.40–1.54 (m, 2H), 1.79–1.85 (m, 2H), 4.25 (t, *J* = 7.3 Hz, 2H), 4.37 (s, 3H), 6.35 (s, 1H), 7.31 (d, *J* = 7.3 Hz, 1H), 7.55–7.62 (m, 2H), 7.65 (t, *J* = 7.7 Hz, 1H), 7.82 (t, *J* = 7.4 Hz, 1H), 8.07 (t, *J* = 7.7 Hz, 1H), 8.12 (d, *J* = 8.1 Hz, 1H), 8.21 (d, *J* = 7.8 Hz, 1H), 8.35 (d, *J* = 8.1 Hz, 2H), 8.58–8.71 (m, 2H); ^13^C-NMR (DMSO-*d*_6_): δ ppm 13.9, 19.7, 29.7, 42.9, 93.6, 106.4, 118.4, 120.0, 123.7, 127.5, 128.9, 129.5, 129.7, 130.4, 133.9, 141.2, 152.1, 157.0; HRMS (ESI): Calcd for C_26_H_25_N_2_^+^ *m*/*z* 365.2012, obsd *m*/*z* 365.1999.

*1-Butyl-2-[(1*,*1*,*3-trimethyl-1*,*3-dihydro-2H-benzo[e]indol-2-ylidene)methyl]benzo[c*,*d]indolium iodide* (**6d**); Yield 0.52 g, 72%; mp 190–192 °C; ^1^H-NMR (DMSO-*d*_6_): δ ppm 0.95 (t, *J* = 7.3 Hz, 3H), 1.45 (q, *J* = 7.3 Hz, 2H), 1.80–1.97 (m, 8H), 3.60 (s, 3H) 4.46 (t, *J* = 7.3 Hz, 2H), 6.43 (s, 1H), 7.60 (t, *J* = 7.5 Hz, 1H), 7.67–7.78 (m, 3H), 7.81 (d, *J* = 7.3 Hz, 1H), 7.84–7.93 (m, 3H), 8.14 (d, *J* = 8.0 Hz, 1H), 8.21 (d, *J* = 8.7 Hz, 1H), 8.35 (d, *J* = 8.2 Hz, 2H); ^13^C-NMR (DMSO-*d*_6_): δ ppm 13.7, 19.7, 25.2, 29.8, 43.8, 53.2, 54.9, 82.9, 110.4, 113.1, 122.9, 123.0, 124.1, 125.8, 127.7, 128.0, 128.6, 129.3, 129.6, 129.8, 130.0, 130.2, 130.3, 132.1, 132.3, 133.6, 140.8, 141.3, 156.5, 181.1; HRMS (ESI): Calcd for C_31_H_31_N_2_^+^ *m*/*z* 431.2482, obsd *m*/*z* 431.2469.

*1-Butyl-2-[(1*,*1*,*3-trimethyl-1*,*3-dihydro-1H-indol-2-ylidene)methyl]benzo[c*,*d]indolium iodide* **(10a**); Yield 0.42 g, 63%; mp 238–240 °C; ^1^H-NMR (DMSO-*d*_6_): δ ppm 0.95 (t, *J* = 7.08 Hz, 3H), 1.42–1.47 (m, 2H), 1.65 (s, 6H), 1.85–1.88 (m, 2H), 3.47 (s, 3H), 4.46 (t, *J* = 7.0 Hz, 2H), 6.31 (s, 1H), 7.44 (t, *J* = 7.3 Hz, 1H), 7.51–7.63 (m, 2H), 7.69–7.85 (m, 4H), 7.88–7.96 (m, 2H), 8.38 (d, *J* = 8.0 Hz, 1H); ^13^C-NMR (DMSO-*d*_6_): δ ppm 13.6, 19.6, 25.6, 29.7, 43.8, 45.7, 51.4, 82.9, 110.9, 113.3, 122.7, 123.2, 123.9, 126.4, 128.6, 129.1, 129.1, 129.5, 130.0, 130.2, 132.5, 140.1, 140.6, 143.9, 156.9, 179.5; HRMS (ESI): Calcd for C_27_H_29_N_2_^+^ *m*/*z* 381.2325, obsd *m*/*z* 381.2313.

*1-Butyl-2-[(3-ethyl-5-methoxy-1*,*1-dimethyl-1*,*3-dihydro-1H-indol-2-ylidene)methyl]benzo[c*,*d] indolium iodide* (**10b**); Yield 0.65 g, 90%; mp 187–189 °C; ^1^H-NMR (DMSO-*d*_6_): δ ppm 0.92 (t, *J* = 7.2 Hz, 3H), 1.14 (t, *J* = 6.7 Hz, 3H), 1.37–1.43 (m, 2H), 1.61 (s, 6H), 1.79–1.83 (m, 2H), 3.85 (s, 3H), 4.20 (q, *J* = 6.3 Hz, 2H), 4.39 (t, *J* = 6.1 Hz, 2H), 6.23 (s, 1H), 7.10 (d, *J* = 9.9 Hz, 1H), 7.40 (s, 1H), 7.58–7.67 (m, 2H), 7.70 (t, *J* = 7.9 Hz, 1H), 7.77–7.87 (m, 2H), 7.87–7.94 (m, 1H), 7.91 (d, *J* = 7.5 Hz, 1H), 8.30 (d, *J* = 8.0 Hz, 2H); ^13^C-NMR (DMSO-*d*_6_): δ ppm 13.3, 13.7, 19.5, 25.1, 29.7, 43.4, 45.6, 51.9, 56.0, 83.4, 109.5, 109.9, 113.8, 115.3, 122.4, 124.1, 127.3, 129.4, 129.5, 129.9, 130.1, 132.1, 134.8, 140.9, 142.8, 154.9, 158.9, 179.4; HRMS (ESI): Calcd for C_29_H_33_N_2_O^+^ *m*/*z* 425.2587, obsd *m*/*z* 425.2576.

*1-Butyl-2-[(5-chloro-3-ethyl-1*,*1-dimethyl-1*,*3-dihydro-1H-indol-2-ylidene)methyl]benzo[c*,*d] indolium iodide* (**10c**); Yield 0.25 g, 34%; mp 152–154 °C; ^1^H-NMR (DMSO-*d*_6_): δ ppm 0.91 (t, *J* = 7.2 Hz, 3H), 1.08 (t, *J* = 6.7 Hz, 3H), 1.37–1.43 (m, 2H), 1.63 (s, 6H), 1.81–1.84 (m, 2H), 4.16 (q, *J* = 6.7 Hz, 2H), 4.47 (t, *J* = 6.3 Hz, 2 H), 6.29 (s, 1H), 7.59 (d, *J* = 8.7 Hz, 1H), 7.67 (d, *J* = 8.7 Hz, 1H), 7.71–7.81 (m, 2H), 7.85–7.95 (m, 3H), 8.10 (d, *J* = 7.3 Hz, 1H), 8.39 (d, *J* = 8.2 Hz, 1H); ^13^C-NMR (DMSO-*d*_6_): δ ppm 13.0, 13.7, 19.4, 25.4, 29.9, 43.8, 45.7, 51.5, 83.8, 111.8, 115.5, 123.5, 123.7, 123.9, 128.4, 128.9, 129.2, 129.6, 130.1, 130.6, 133.2, 140.5, 140.8, 142.7, 156.8, 179.1; HRMS (ESI): Calcd for C_28_H_30_N_2_Cl^+^ *m*/*z* 429.2092, obsd *m*/*z* 429.2083.

### 3.3. Stock Solutions for Optical Measurements

Stock solutions were prepared by weighing the solid of each individual compound on a 5-digit analytical balance and adding solvent via class A volumetric pipette to make a 1.0 mM solution. The vials were vortexed for 20 s and then sonicated for 5 min to ensure complete dissolution. When not in use, the stock solutions were stored in a dark at 4 °C. For emission spectra in methanol/glycerol solutions the concentrations were prepared via the dilution of the stock solution in methanol followed by the addition of the appropriate volume of glycerol to achieve the desired concentrations.

### 3.4. Method of Determining Absorbance and Fluorescence

Stock solutions were used to prepare five dilutions of dyes with concentrations ranging from 5 to 25 μM using a class A volumetric pipette in order to maintain absorption between 0.1 and 1.0. The dye solutions were diluted ten-fold for fluorescence in order to minimize inner filter effect. The absorption spectra of each sample were measured in duplicate from 400 to 750 nm. Aggregation of **6b** was ruled out by measuring absorption at different concentrations (Figure S2G). Dye **6b** was tested for solvatochromic changes in absorption by dissolving the dye in five different solvents (ethanol, dimethyl formamide, dichloromethane, acetonitrile, and aqueous tris buffer) to observe any change in λ_max_ (Supplementary Materials Figure S2H). The emission spectra of each sample were measured in duplicate with a 530 nm excitation wavelength and slit widths of 5 nm for both excitation and emission. Emission spectra were corrected automatically by our developed method file used for reading the spectrofluorometer.

### 3.5. Computational Methods

The structure of each compound was first optimized using the TD-DFT method with the hybrid exchange-correlation functional, B3LYP/6-31G* basis set using *SPARTAN ‘14* (Wavefunction, Inc., Irvine, CA, USA) [63]. The torsional angles from the quaternary nitrogen to the α-carbon on the alternate heterocycle were restricted to 0° to get the calculated absorbance values, LUMO and HOMO orbitals, and electrostatic potential maps. The calculated LUMO and HOMO orbitals were obtained using a restricted hybrid HF-DFT SCF calculation performed with B3LYP/6-31G* basis set. The electrostatic potential maps were investigated for the optimized structures at HF/6-31G*. DNA docking studies were achieved using AutoDockTools 1.5.6 (Scripps Research Institute, La Jolla, CA, USA). Results of DNA docking study with dye **6b** under constraints were obtained by making all bonds within the dye to be non-rotatable and planar [64,65]. Polar and aromatic hydrogens were added to the DNA using GROMACS package [66] using GROMOS 53A6 force field [67] and Gasteiger Marsili charges [68]. A 78 × 70 × 64 grid box with a resolution of 0.375 Å was created encompassing the entire DNA using module AutoGrid 4.0. Dye **6b** was then added and simulations were preformed using Genetic Algorithm (GA).

### 3.6. DNA Binding Studies

A stock solution of **6b** (1 × 10^−4^ M) and ct-DNA type 1 (7.5 × 10^−3^ M) were prepared in ethanol and Tris-HCl buffer solution, respectively. Fluorescence titration with ct-DNA concentrations (0–200 mM) were made by mixing 35 μL **6b** solution with Tris-HCl buffer solution with and without ct-DNA to a total volume of 3500 μL in a fluorescence cuvette to make working solutions of 10 μM **6b**. Fluorescence spectra were measured in duplicate with excitation at 520 nm and slit widths of 10 nm for both excitation and emission.

## 4. Conclusions

A series of seven monomethine cyanines were synthesized in good yield with red-shifted absorbance properties in comparison to previously synthesized monomethine cyanine dyes. Although the benz[*c*,*d*]indolium containing monomethine cyanine dyes in this report are non-fluorescent in free flowing solvent, when the dyes are in a viscous environment their fluorescence becomes observable due to the restricted ability to rotate around the methine bridge. Computational methods outlined above were shown to be useful as a predictive tool for determining their optical properties. Dye **6b** was chosen as a representative example for DNA binding studies and was shown to bind DNA with an observable increase in fluorescence. Computational studies suggest it is binding the minor groove. Utilizing the described techniques these dyes could be developed as potential biological probes. Future studies will investigate how the different heterocycles and substituents affect binding to biological targets.

## Supplementary Materials

Supplementary materials can be accessed at: http://www.mdpi.com/1420-3049/21/1/23/s1.
