# Supplementary material for: Benz[c,d]indolium-containing Monomethine Cyanine Dyes: Synthesis and Photophysical Properties
Source: Molecules. 2015 Dec 24;21(1):23. doi: 10.3390/molecules21010023 (PMC6274575; doi:10.3390/molecules21010023)
Supplement: Supplementary file 1 [file molecules-21-00023-s001.pdf]

Supplementary Materials: Benz[*c*,*d*]indolium Containing Monomethine Cyanine Dyes: Synthesis and Photophysical Properties

Eduardo Soriano, Cory Holder, Andrew Levitz and Maged Henary

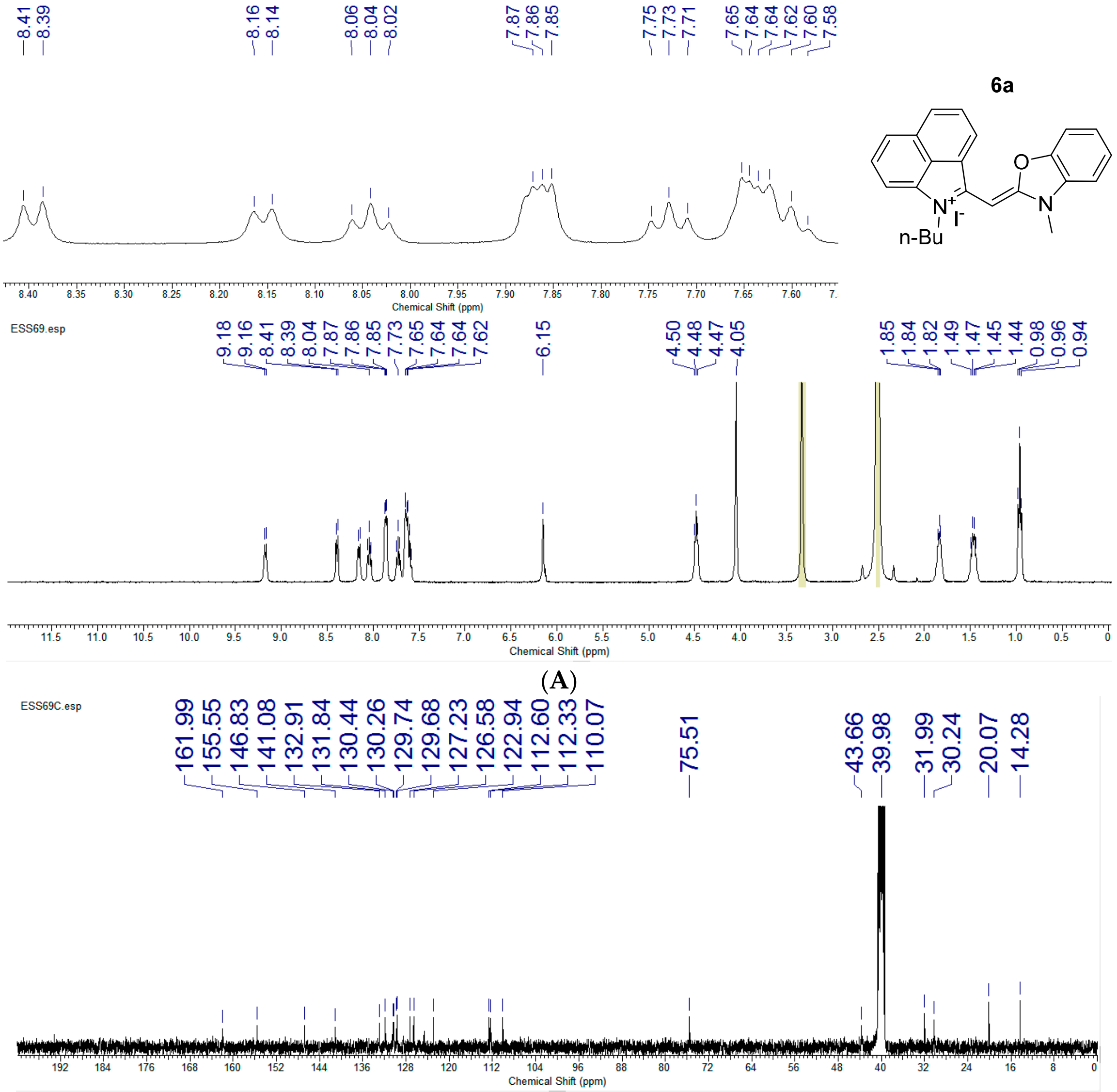

(**B**)

Figure S1. *Cont.*

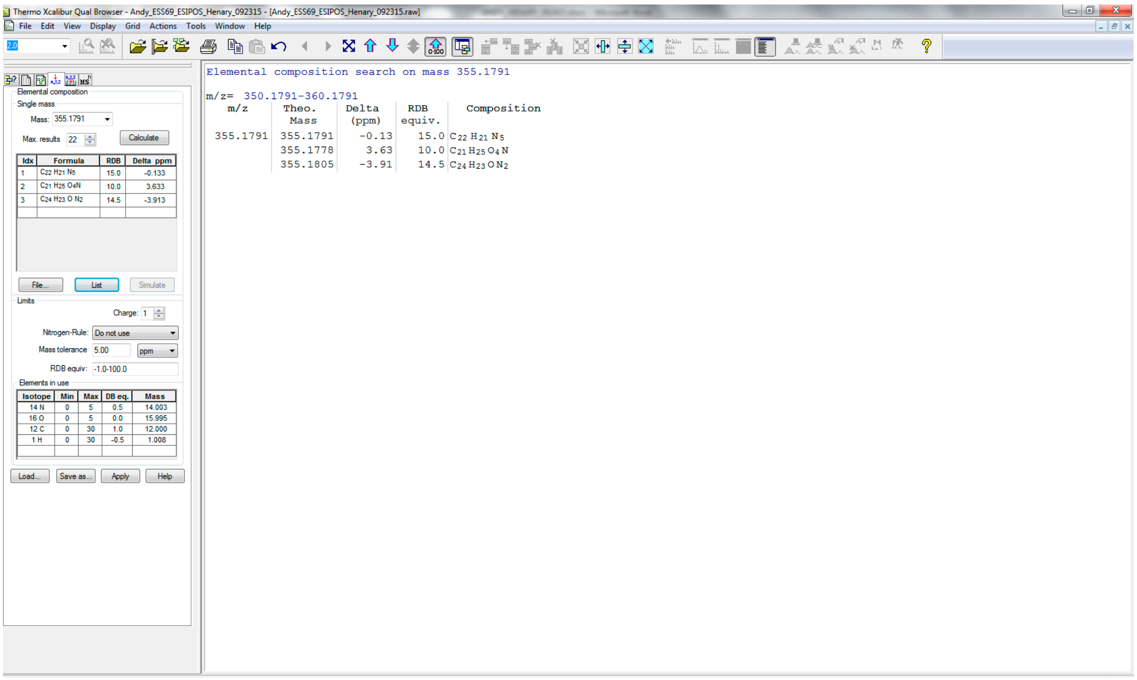

(C)

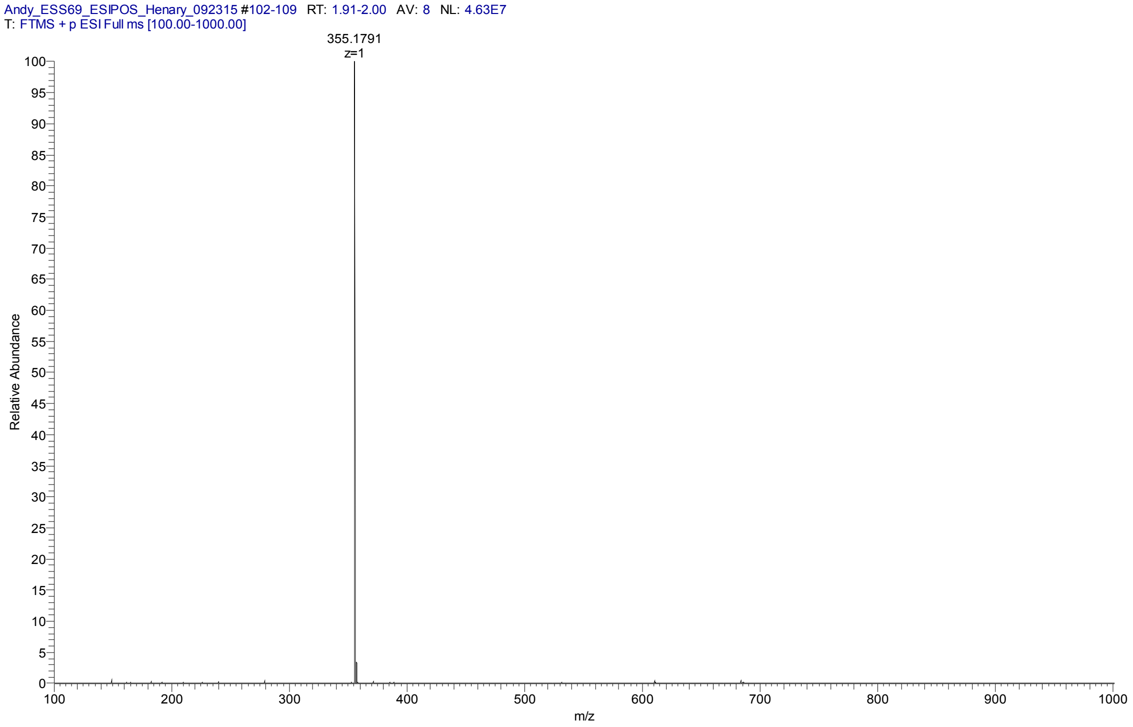

(D)

Figure S1. *Cont.*

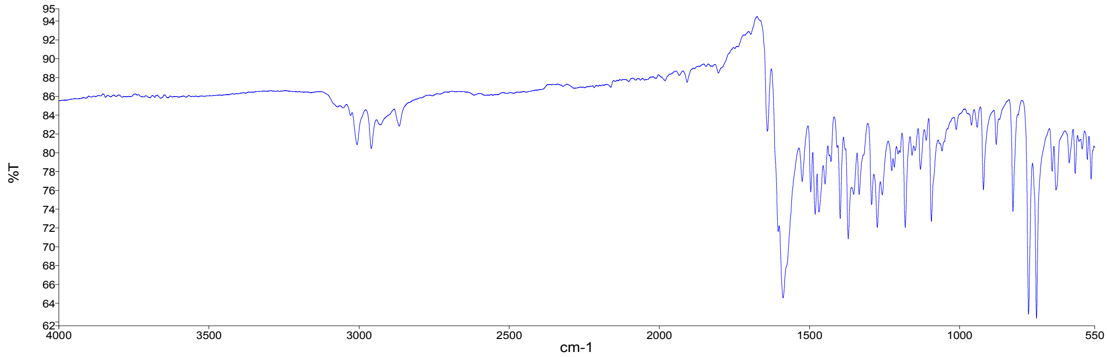

(**E**)

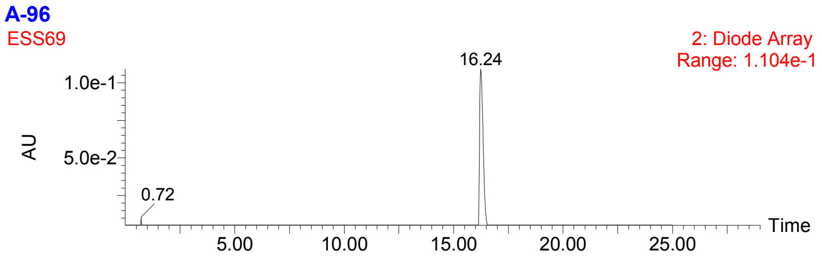

(**F**)

Figure S1. (**A**) ^{1}H-NMR (400 MHz, DMSO-*d*6) 25 °C; (**B**) ^{13}C-NMR (100 MHz, DMSO-*d*6) 25 °C; (**C**) HRMS; (**D)** HRMS; (**E**) IR; (**F**) HPLC data was obtained using a Waters 2487 dual detector wavelength absorption detector with wavelengths set at 260 and 600 nm. The column used in LC was a Waters Delta-Pak 5 µM 100 Å 3.9 × 150 mm reversed phase C18 column, with a flow rate of 1 mL/min employing a 5%–100% acetonitrile/water/0.1% formic acid gradient.

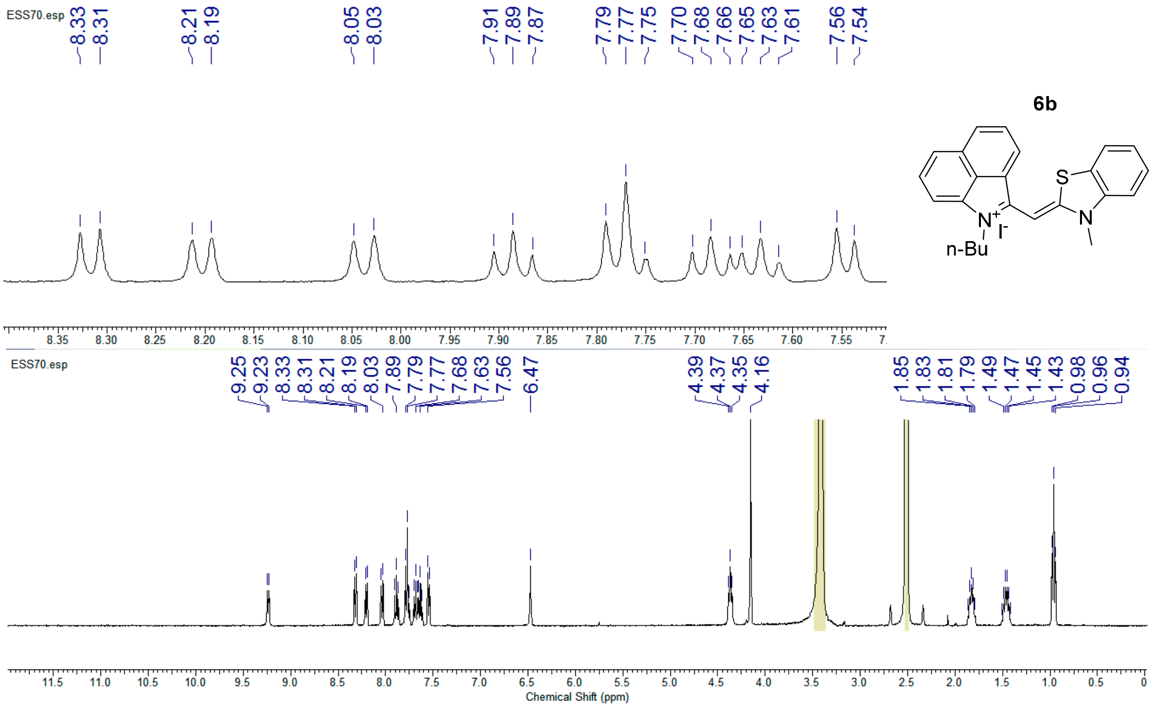

(**A**)

Figure S2. *Cont.*

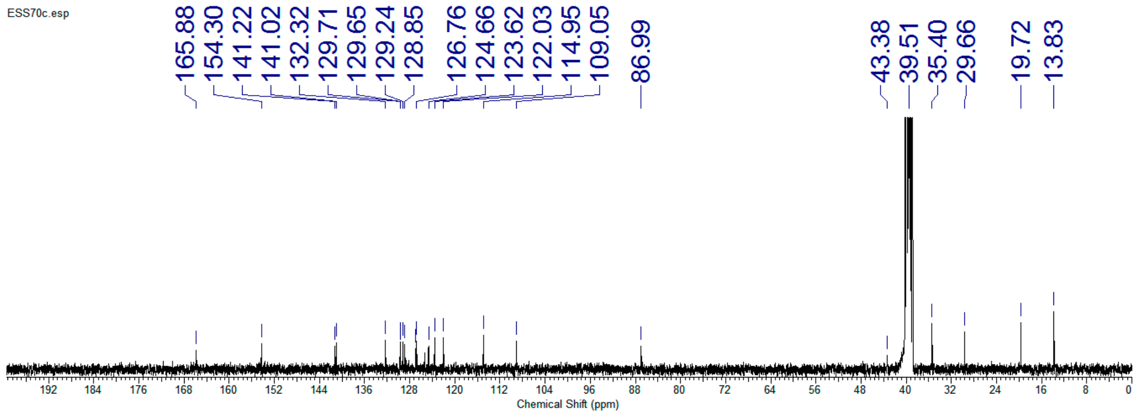

(**B**)

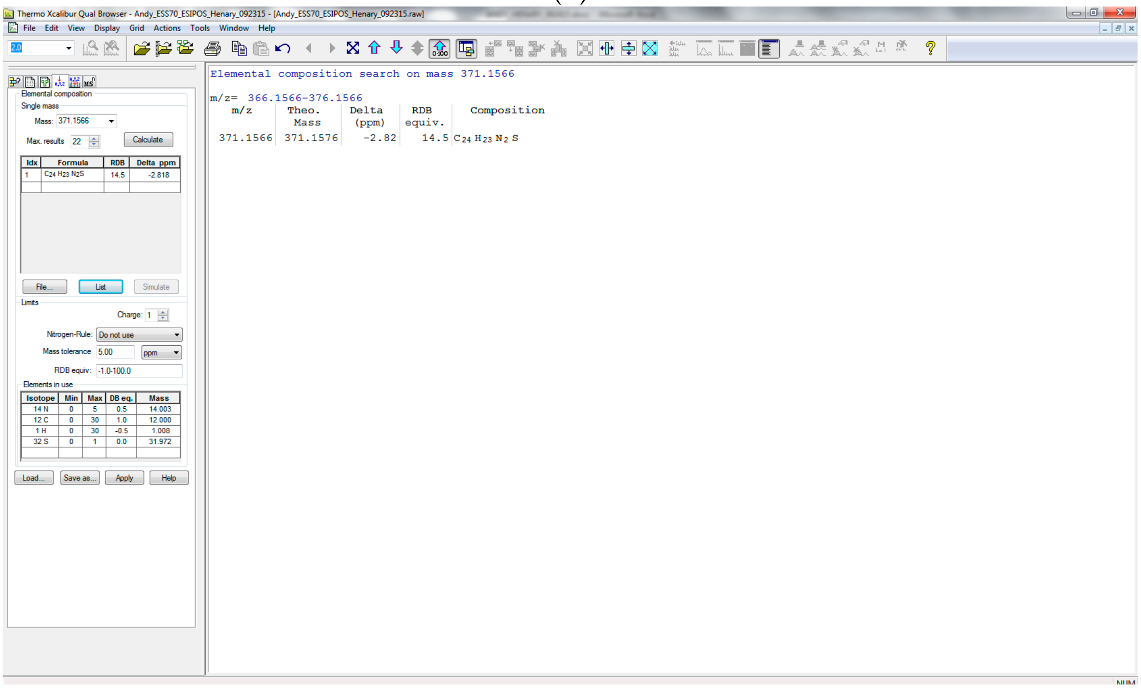

(**C**)

Figure S2. *Cont.*

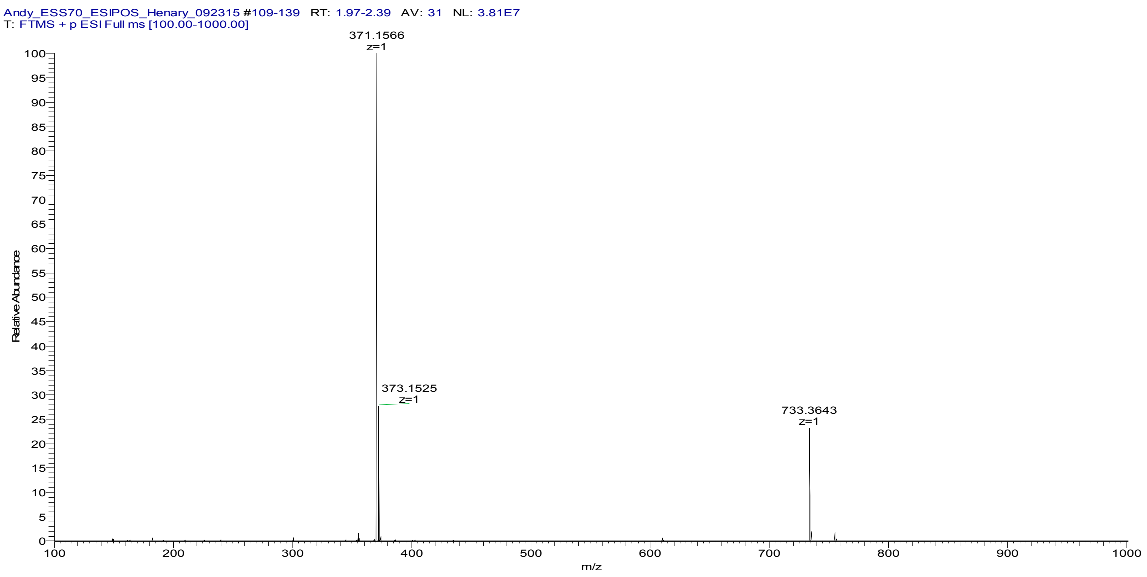

(**D**)

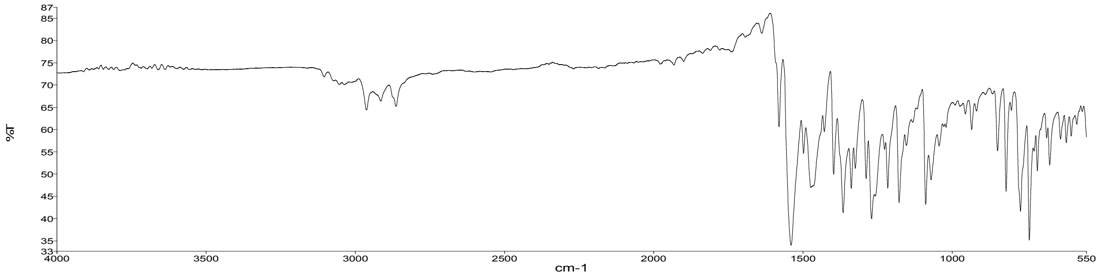

(**E**)

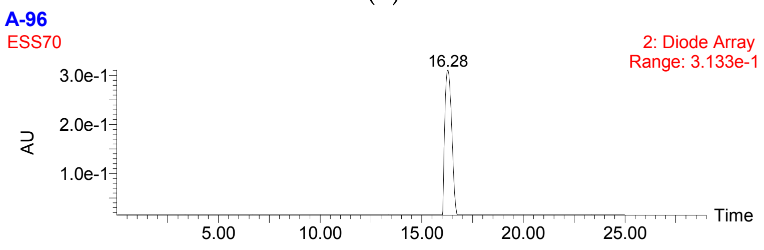

(**F**)

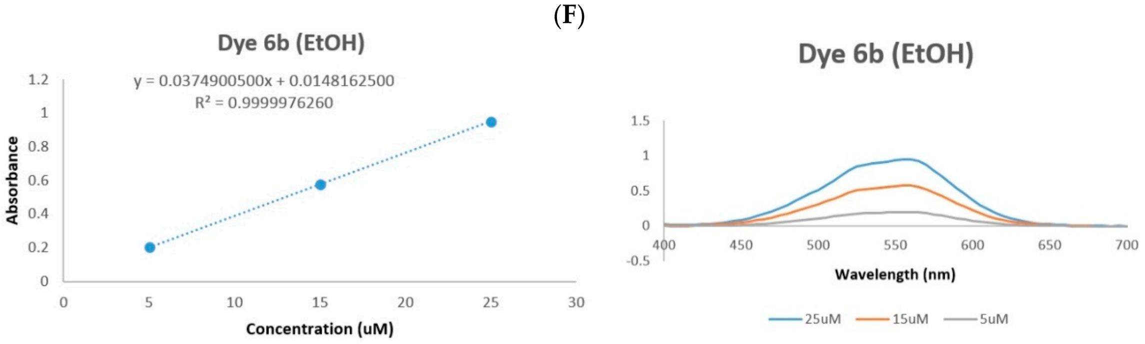

μM Dye 6b	560nm	520nm	ratio
25	0.952401	0.7903	1.205113248
15	0.5765	0.47902	1.20349881
5	0.2026	0.1675	1.209552239

(**G**)

Figure S2. *Cont.*

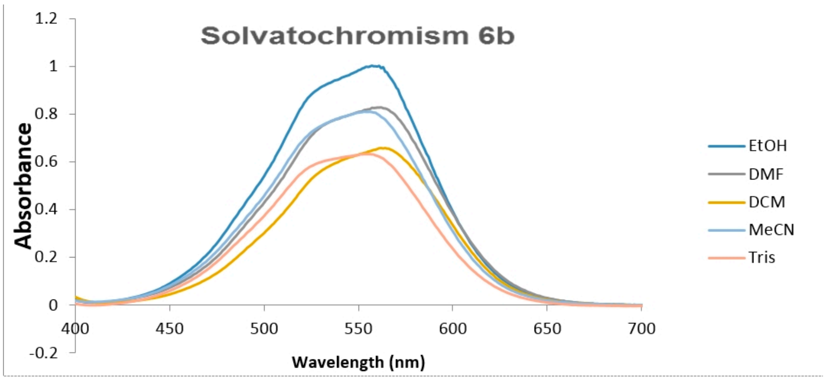

(**H**)

Figure S2. (**A**) ^{1}H-NMR (400 MHz, DMSO-d_6) 25 °C; (**B**) ^{13}C-NMR (100 MHz, DMSO-d_6) 25 °C; (**C**) HRMS; (**D)** HRMS; (**E**) IR; (**F**) HPLC data was obtained using a Waters 2487 dual detector wavelength absorption detector with wavelengths set at 260 and 600 nm. The column used in LC was a Waters Delta-Pak 5 μM 100 Å 3.9 × 150 mm reversed phase C18 column, with a flow rate of 1 mL/min employing a 5%–100% acetonitrile/water/0.1% formic acid gradient; (**G**) Aggregation of 6b was ruled out by measuring absorption at different concentrations. The ratio between the absorption at 560 nm and 520 nm remains the same at high and low concentrations; (**H**) Dye 6b was tested for solvatochromic changes in absorption by dissolving the dye 6b in 5 different solvents (ethanol, dimethyl formamide, dichloromethane, acetonitrile, and aqueous tris buffer) to observe any change in lmax. Less than 5 nm change in lmax was observed. Such a small shift suggests that the distribution of the ground state dye is virtually unaffected by the solvent polarity.

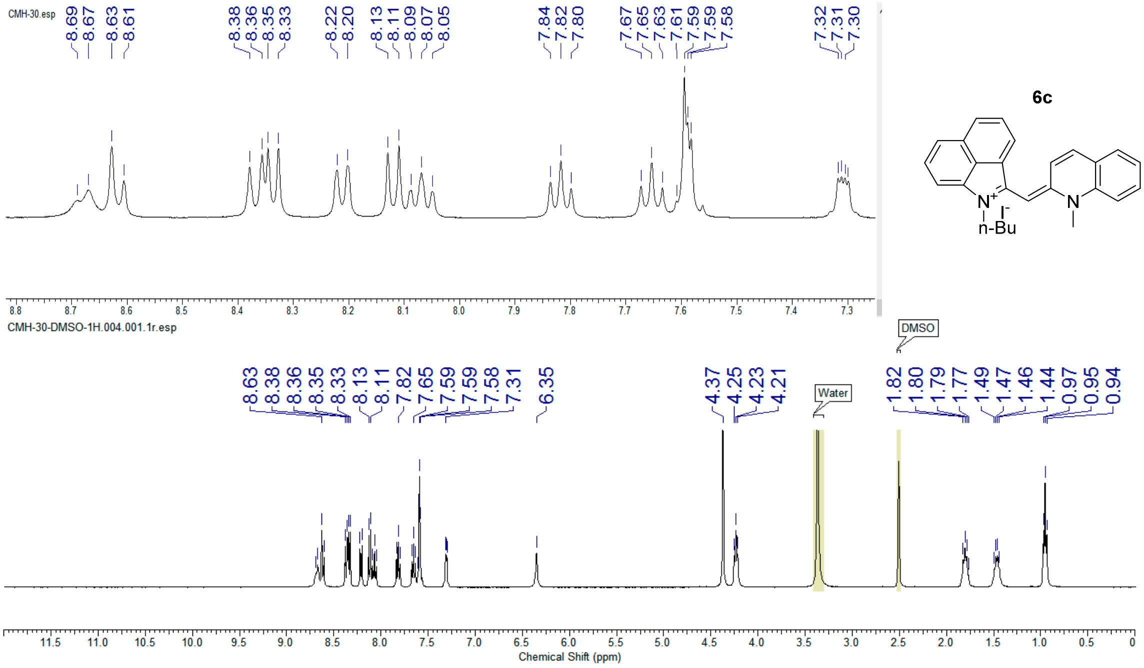

(**A**)

Figure S3. *Cont.*

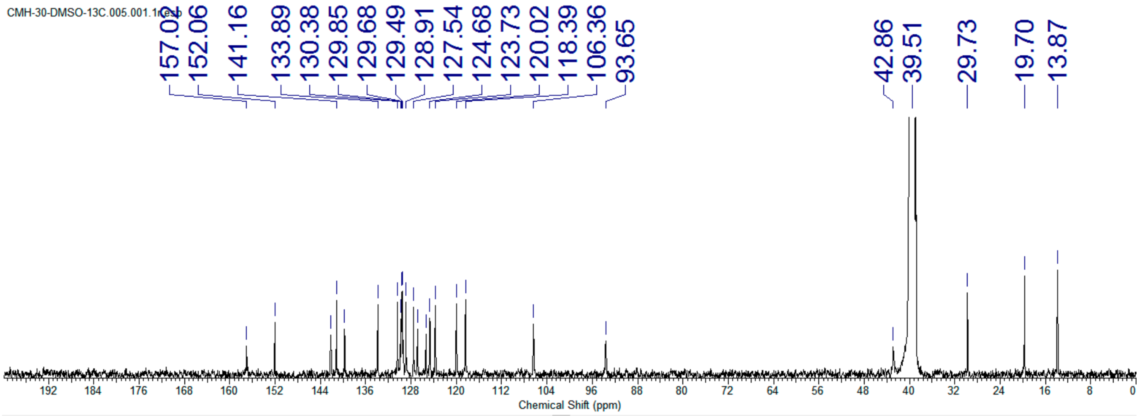

(**B**)

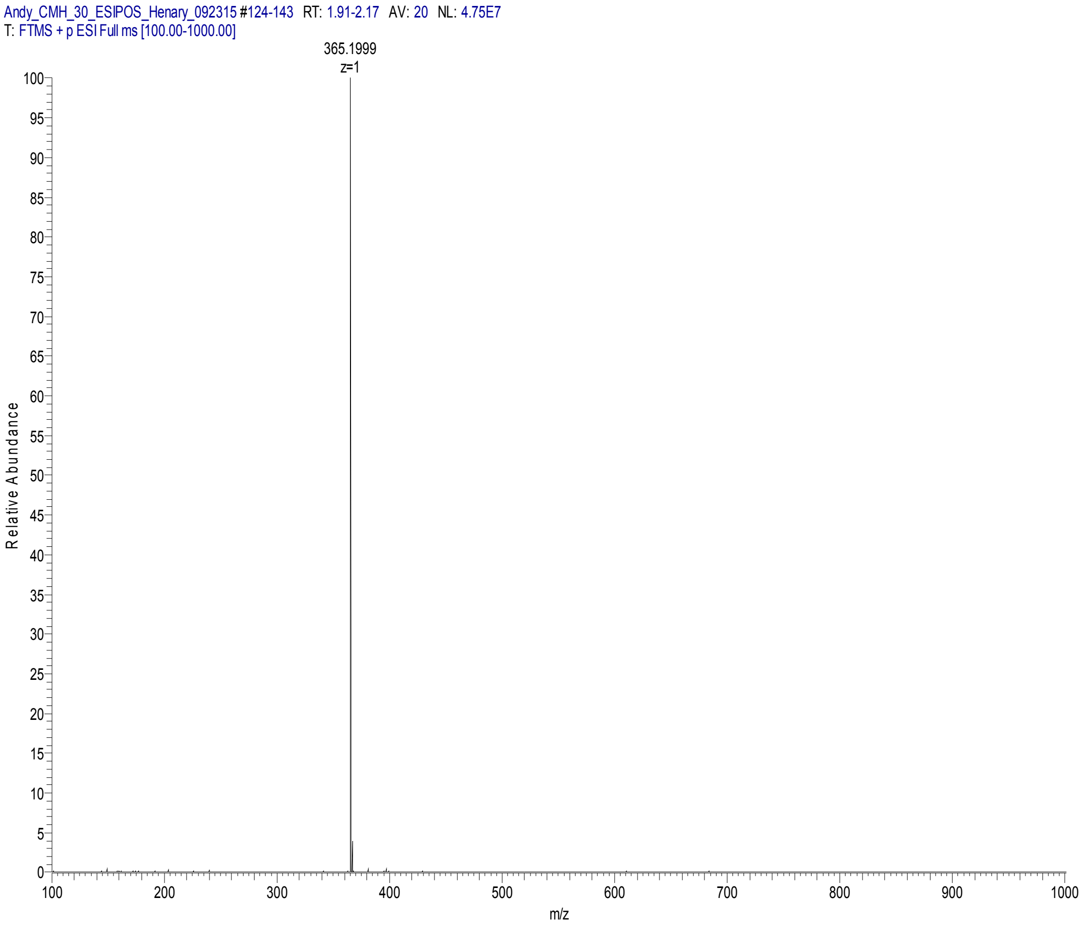

(**C**)

Figure S3. (**A**) ^{1}H-NMR (400 MHz, DMSO-d_6) 25 °C; (**B**) ^{13}C-NMR (100 MHz, DMSO-d_6) 25 °C; (**C**) HRMS.

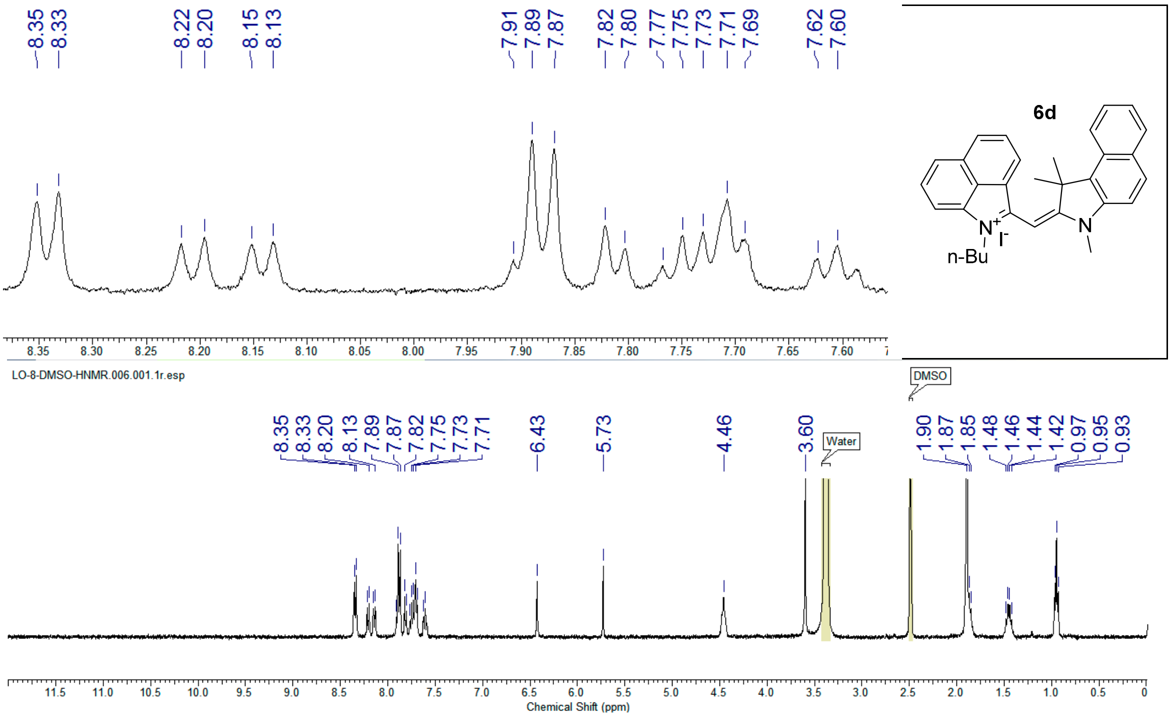

(**A**)

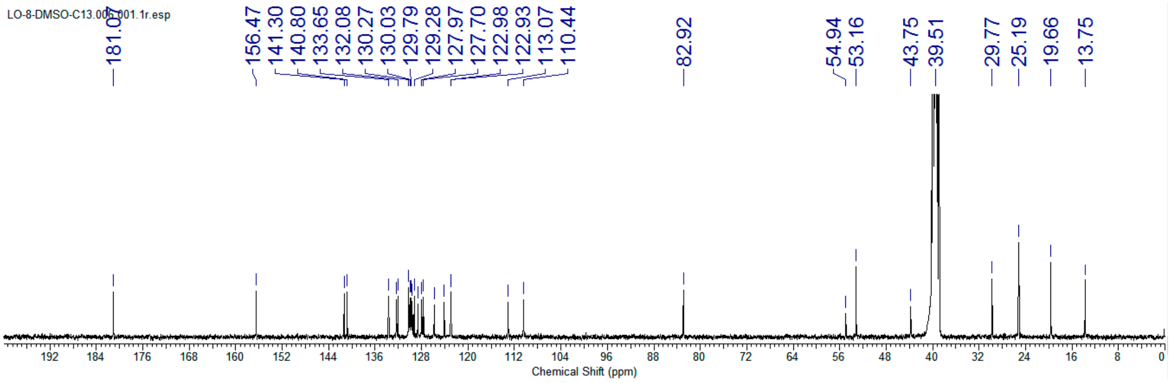

(**B**)

Figure S4. *Cont.*

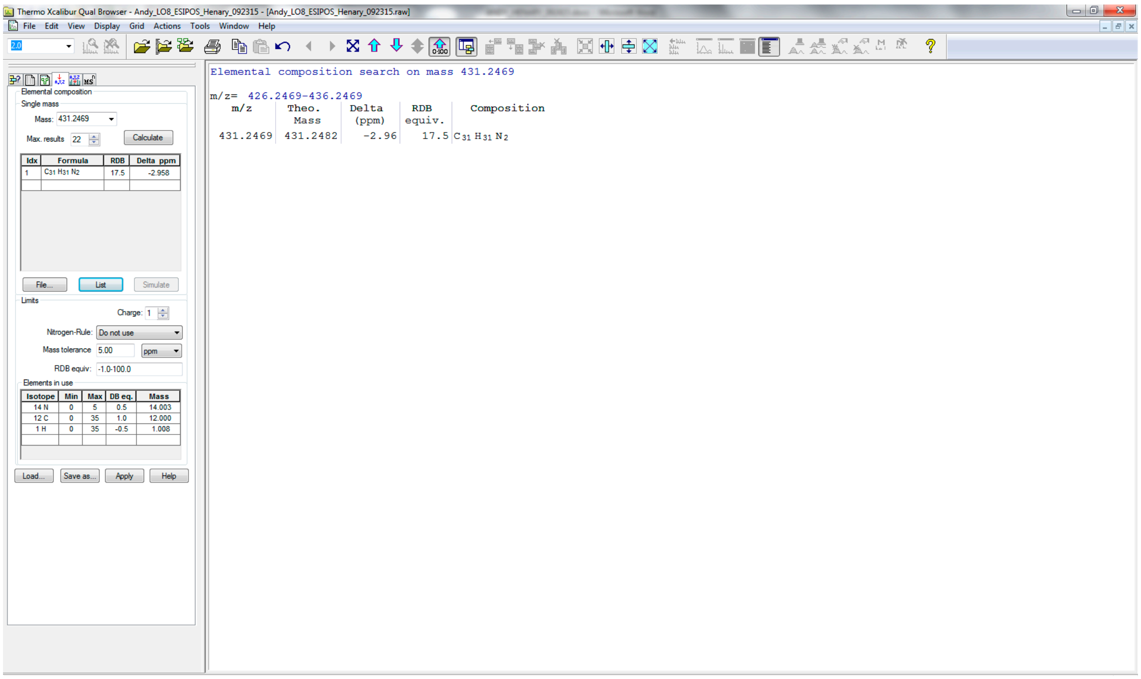

(**C**)

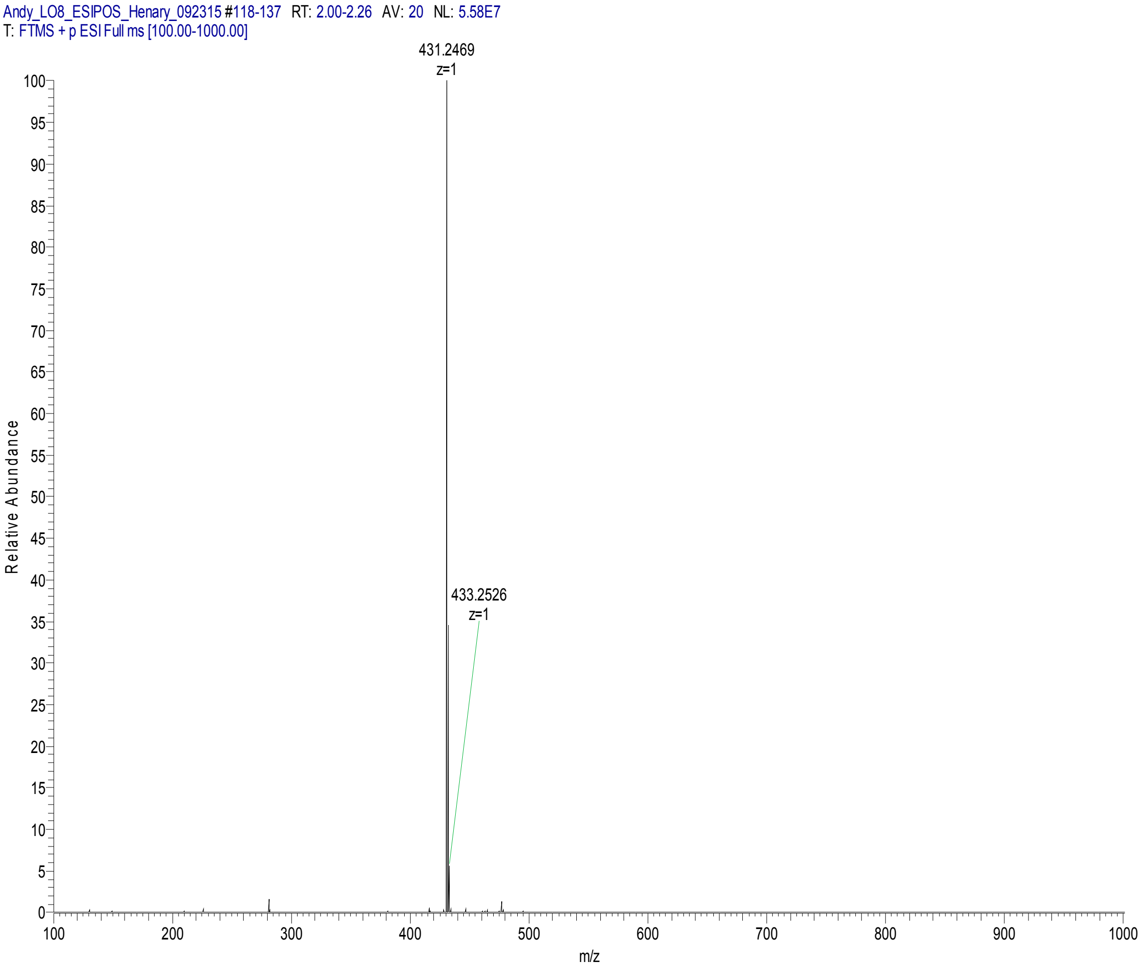

(**D**)

Figure S4. *Cont.*

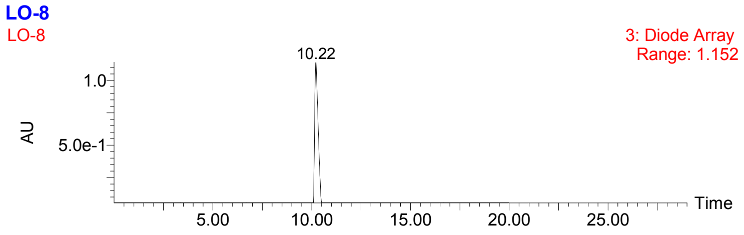

(**E**)

Figure S4. (**A**) ^{1}H-NMR (400 MHz, DMSO-d_6) 25 °C; (**B**) ^{13}C-NMR (100 MHz, DMSO-d_6) 25 °C; (**C**) HRMS; (**D)** HRMS; (**E**) HPLC data was obtained using a Waters 2487 dual detector wavelength absorption detector with wavelengths set at 260 and 600 nm. The column used in LC was a Waters Delta-Pak 5 µM 100 Å 3.9 × 150 mm reversed phase C18 column, with a flow rate of 1 mL/min employing a 5%–100% acetonitrile/water/0.1% formic acid gradient; Caption.

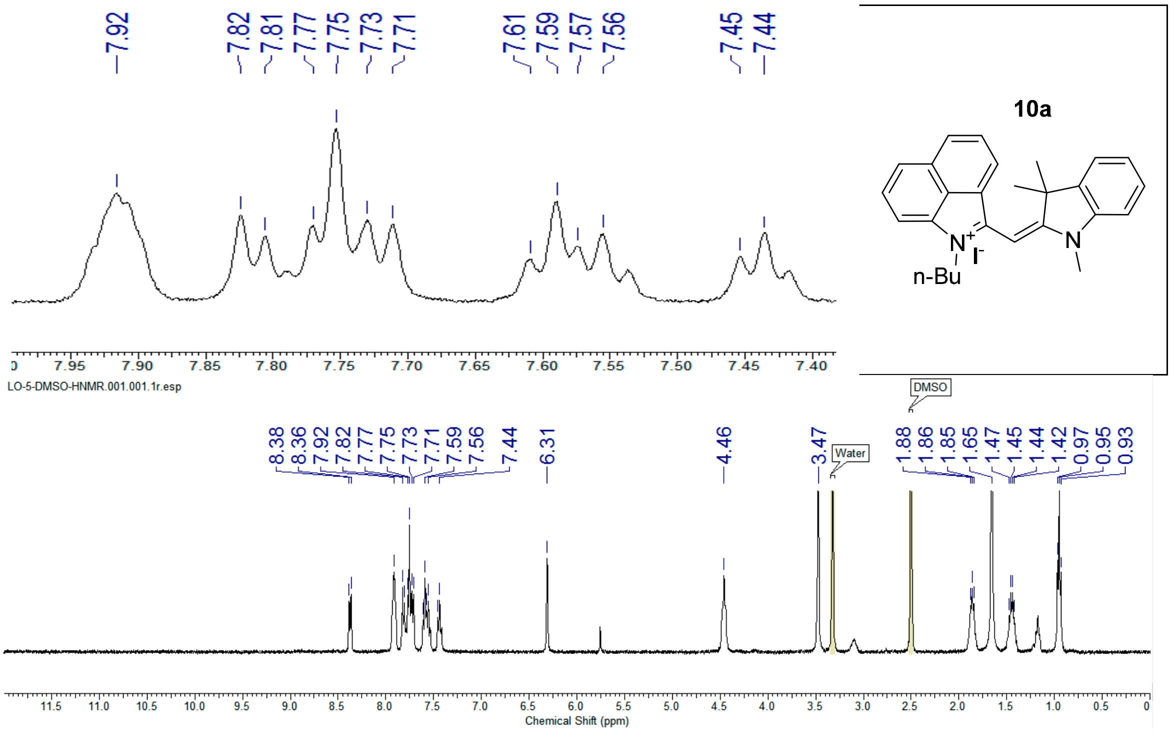

(**A**)

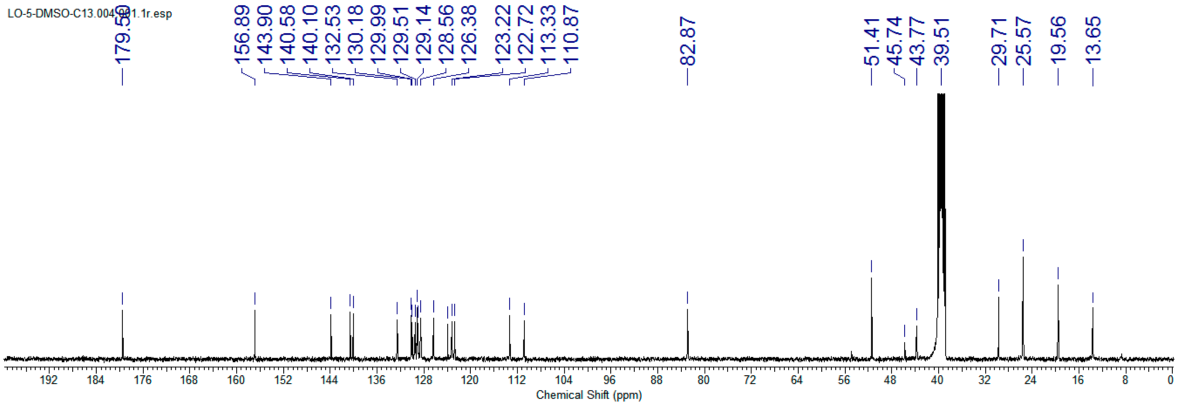

(**B**)

Figure S5. *Cont.*

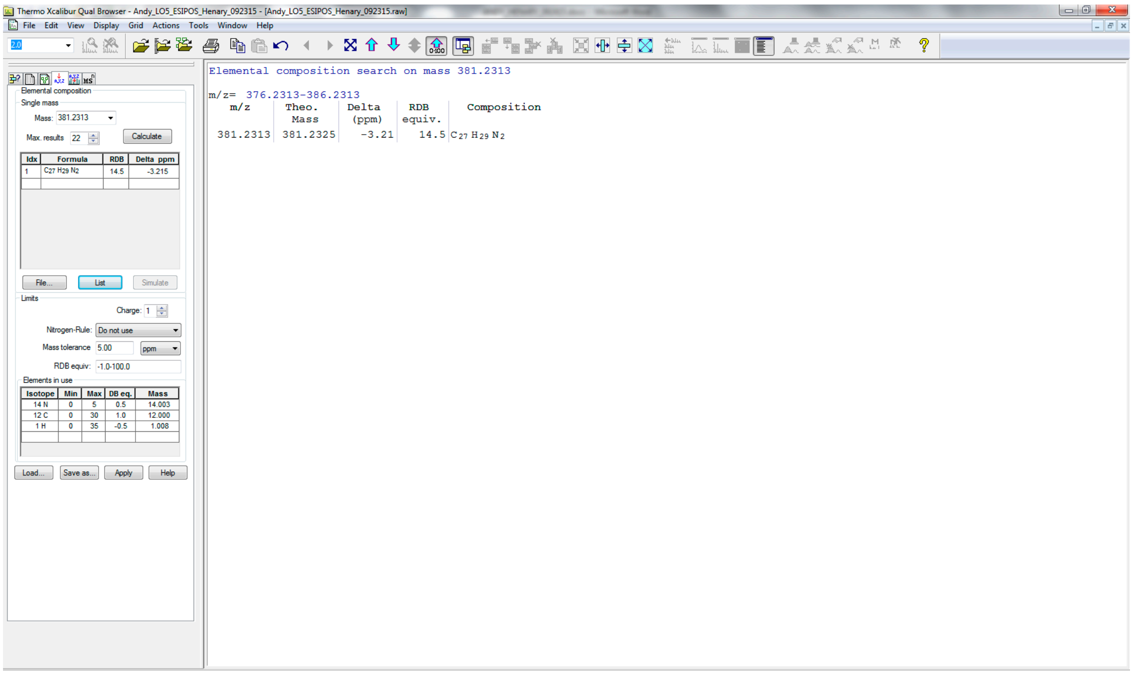

(**C**)

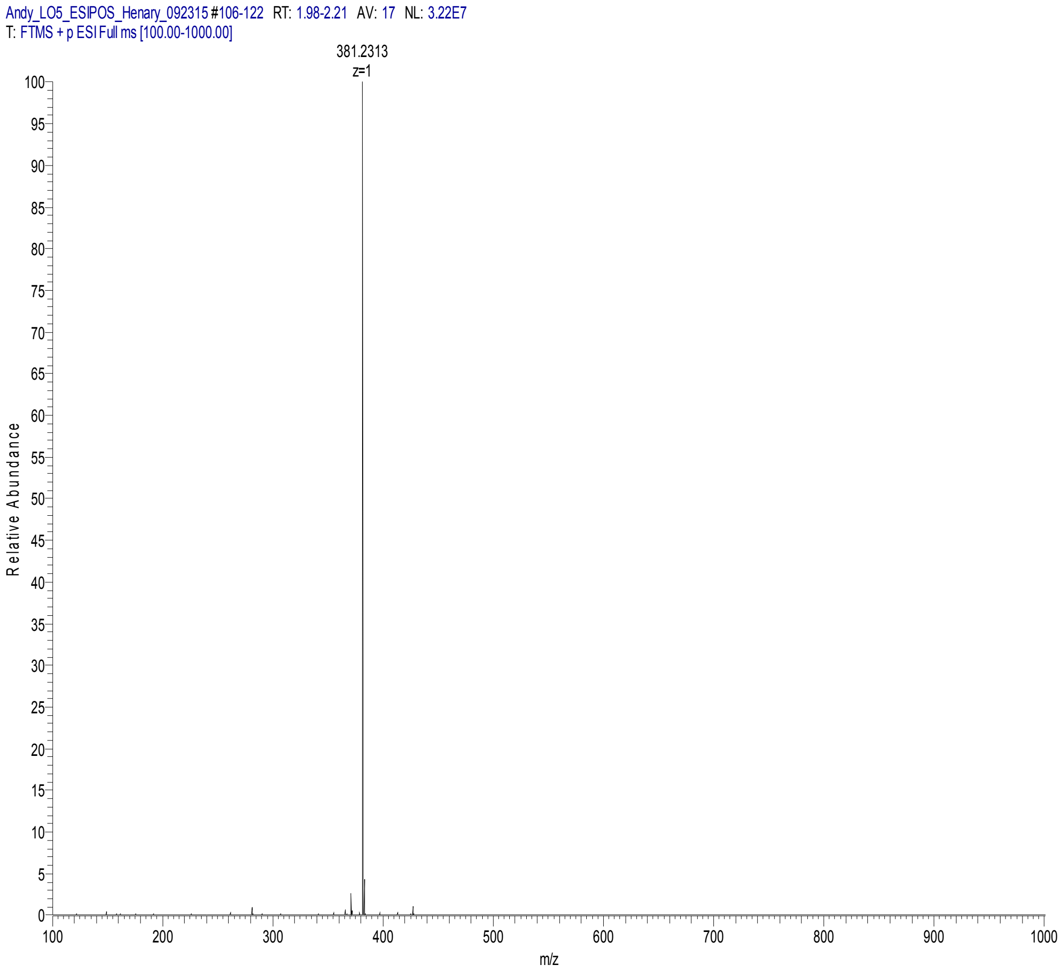

(**D**)

Figure S5. *Cont.*

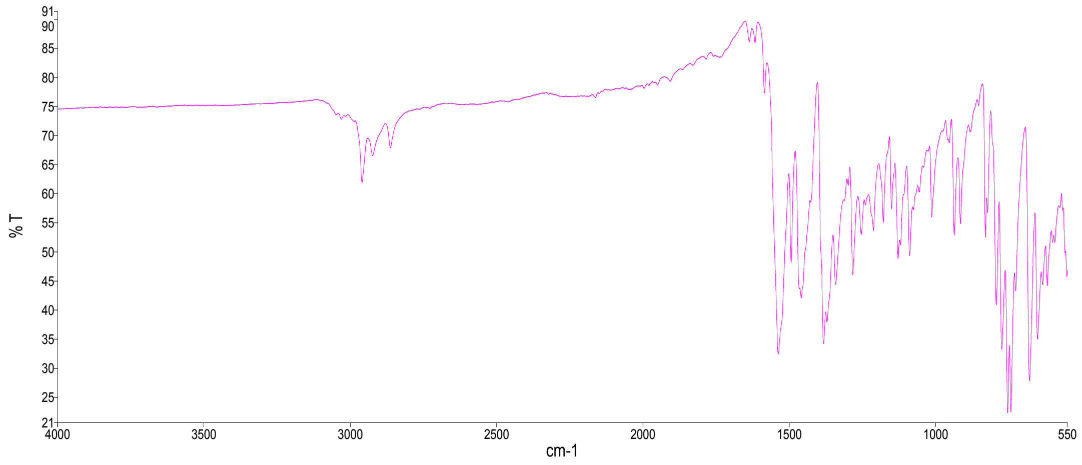

(**E**)

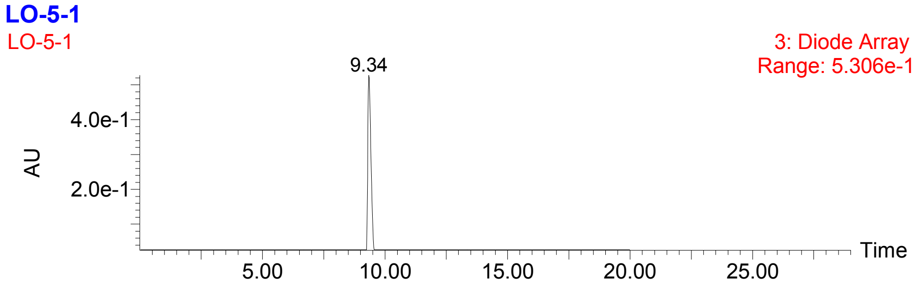

(**F**)

Figure S5. (**A**) ^{1}H-NMR (400 MHz, DMSO-d_6) 25 °C; (**B**) ^{13}C-NMR (100 MHz, DMSO-d_6) 25 °C; (**C**) HRMS; (**D)** HRMS; (**E**) IR; (**F**) HPLC data was obtained using a Waters 2487 dual detector wavelength absorption detector with wavelengths set at 260 and 600 nm. The column used in LC was a Waters Delta-Pak 5 μM 100 Å 3.9 × 150 mm reversed phase C18 column, with a flow rate of 1 mL/min employing a 5%–100% acetonitrile/water/0.1% formic acid gradient;

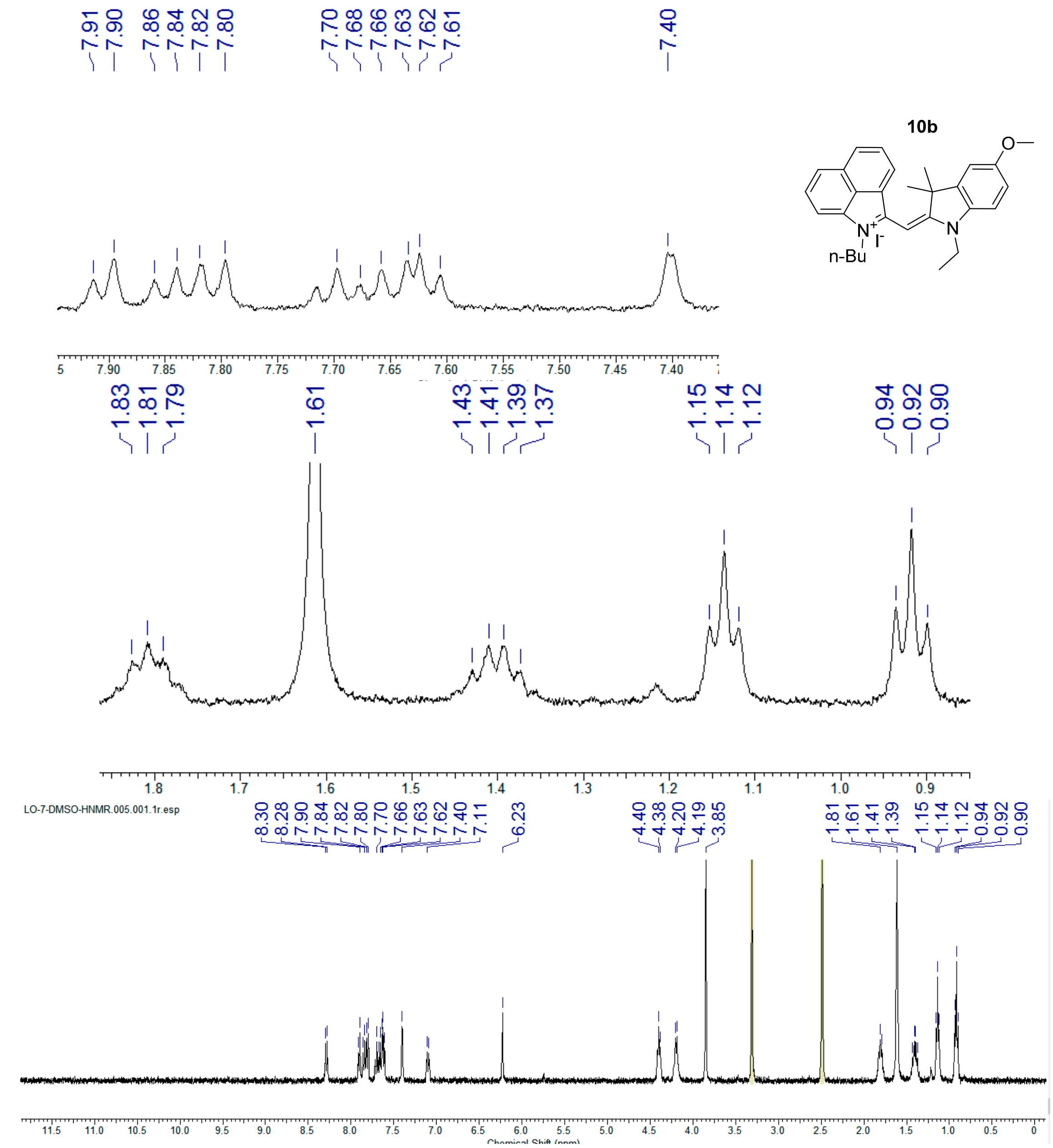

(**A**)

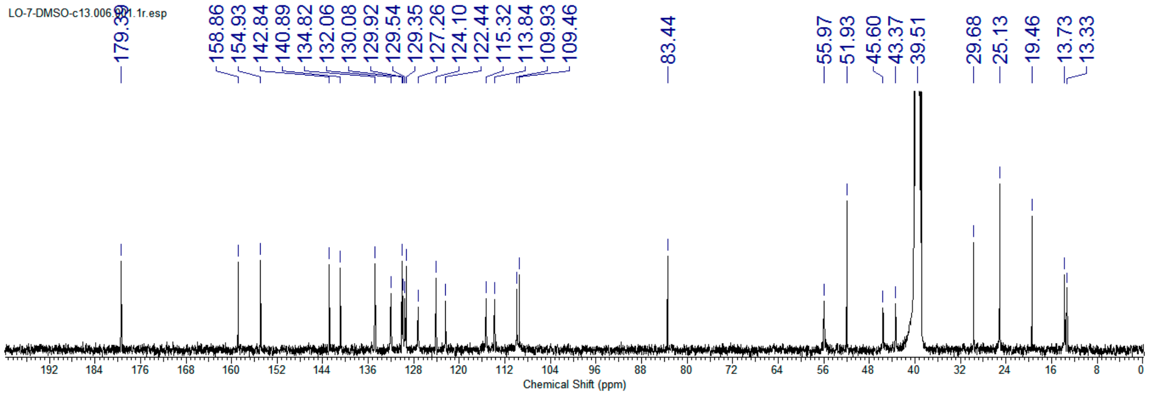

(**B**)

Figure S6. *Cont.*

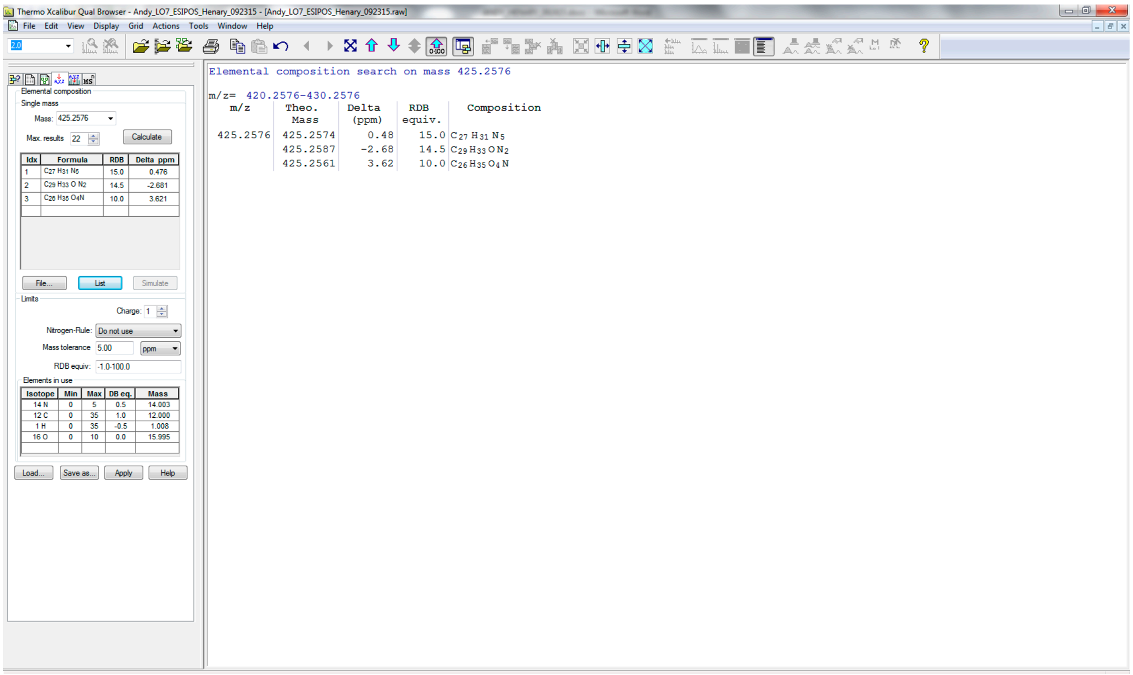

(**C**)

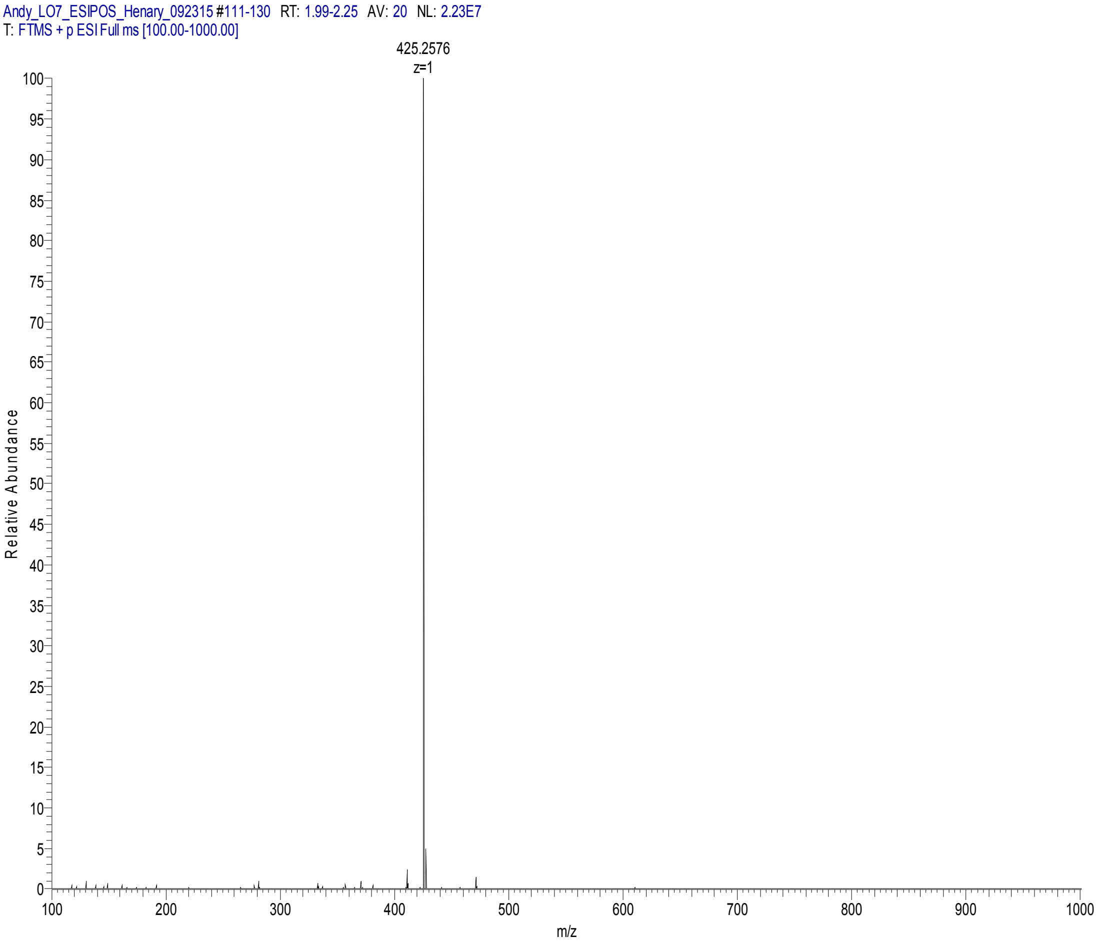

(**D**)

Figure S6. *Cont.*

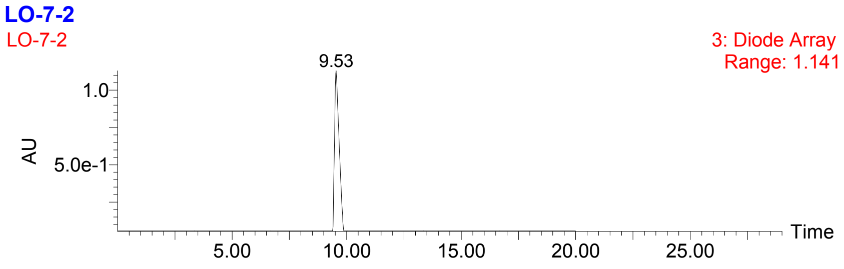

(**E**)

Figure S6. (**A**) ^{1}H-NMR (400 MHz, DMSO-d_6) 25 °C; (**B**) ^{13}C-NMR (100 MHz, DMSO-d_6) 25 °C; (**C**) HRMS; (**D)** HRMS; HPLC data was obtained using a Waters 2487 dual detector wavelength absorption detector with wavelengths set at 260 and 600 nm. The column used in LC was a Waters Delta-Pak 5 μM 100 Å 3.9 × 150 mm reversed phase C18 column, with a flow rate of 1 mL/min employing a 5%–100% acetonitrile/water/0.1% formic acid gradient;

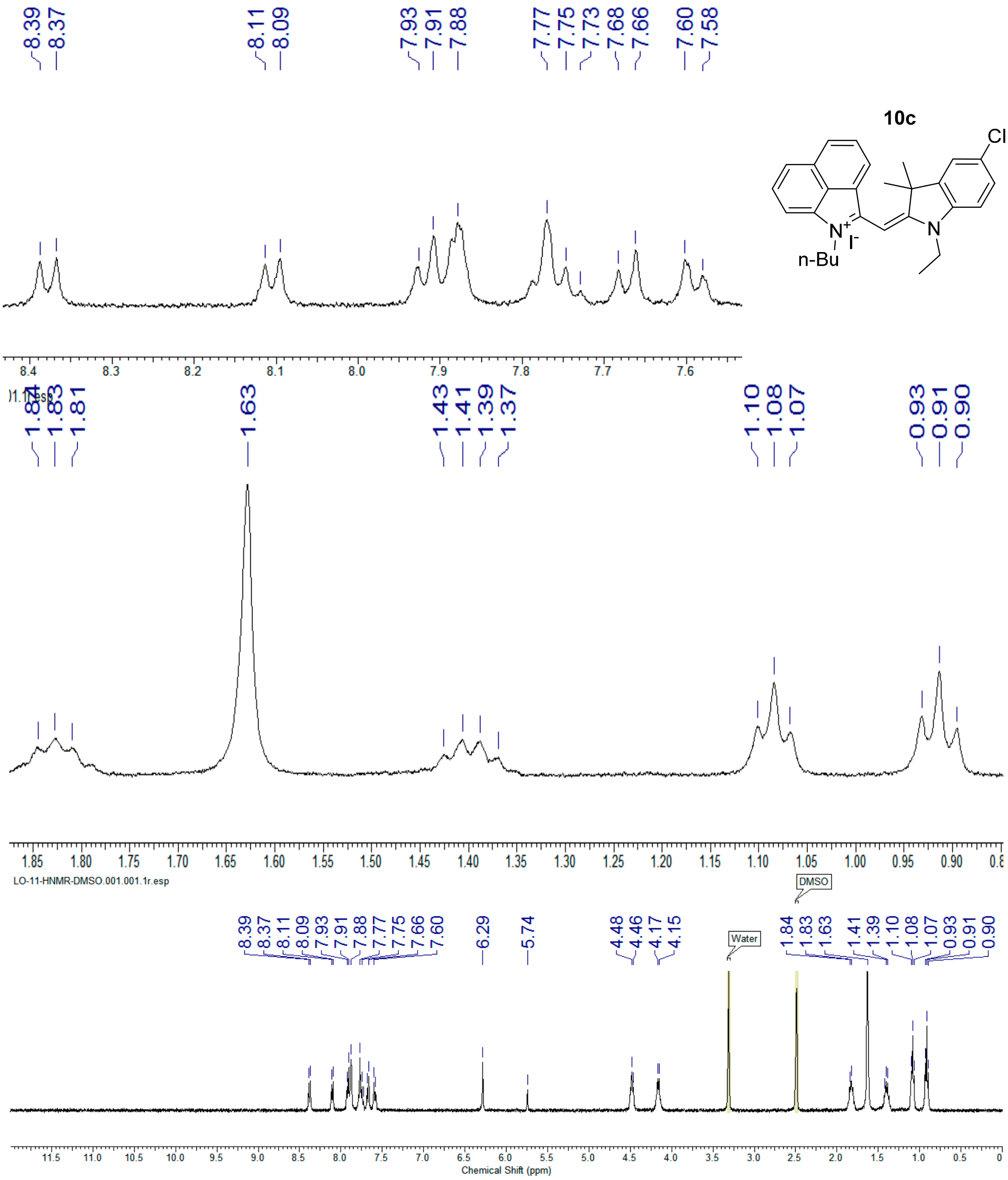

(**A**)

Figure S7. *Cont.*

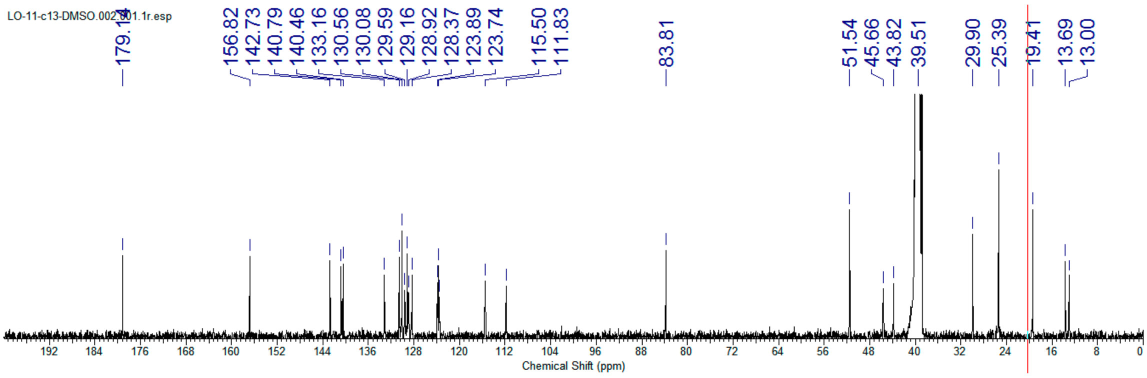

(**B**)

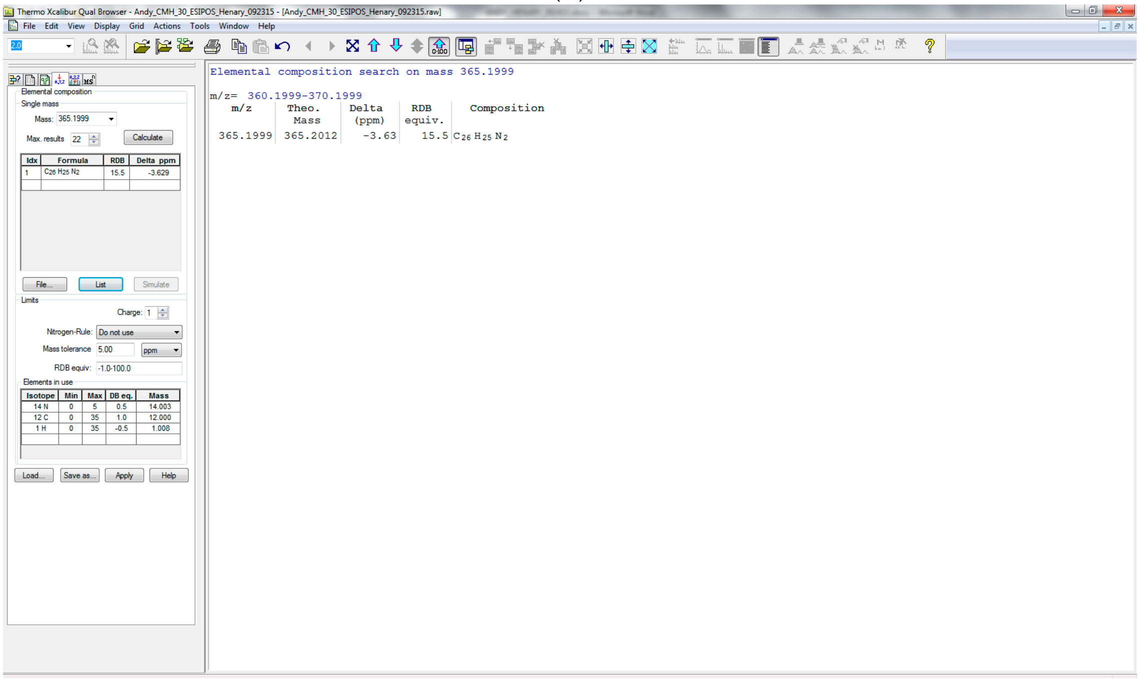

(**C**)

Figure S7. *Cont.*

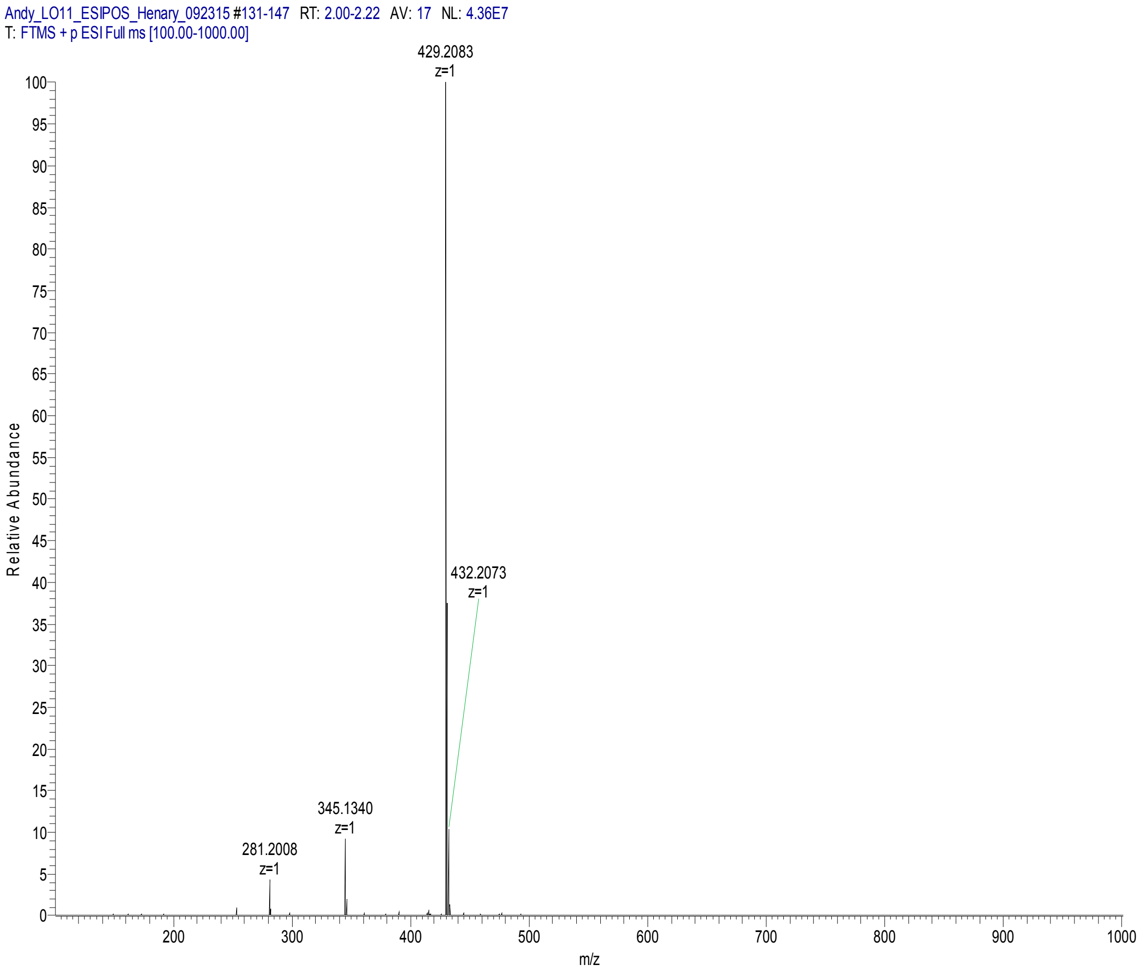

(**D**)

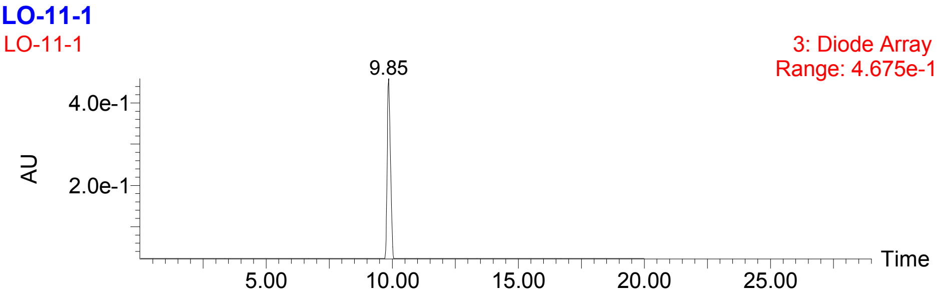

(**E**)

Figure S7. (**A**) ^{1}H-NMR (400 MHz, DMSO-d_6) 25 °C; (**B**) ^{13}C-NMR (100 MHz, DMSO-d_6) 25 °C; (**C**) HRMS; (**D)** HRMS; (**E**) HPLC data was obtained using a Waters 2487 dual detector wavelength absorption detector with wavelengths set at 260 and 600 nm. The column used in LC was a Waters Delta-Pak 5 μM 100 Å 3.9 × 150 mm reversed phase C18 column, with a flow rate of 1 mL/min employing a 5%–100% acetonitrile/water/0.1% formic acid gradient;

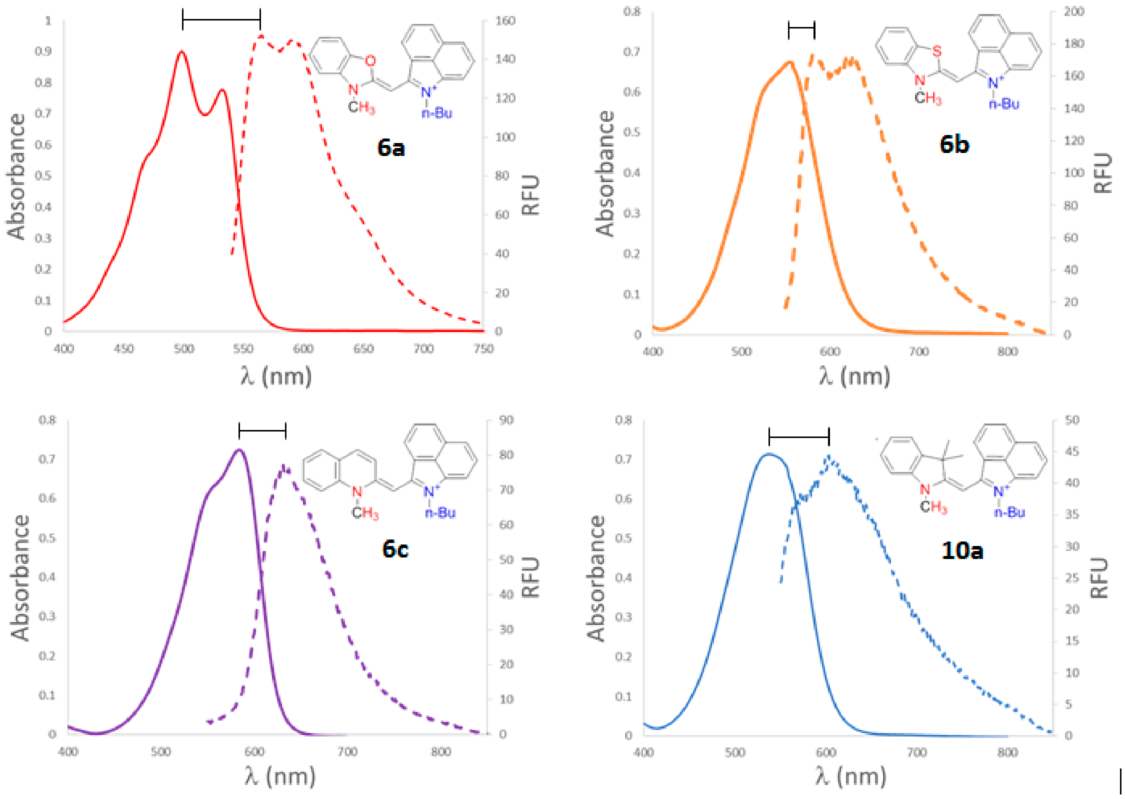

Figure S8. Absorbance (solid lines) and emission (dashed lines) in methanol/glycerol 9/1 spectra at 20 µM.
